# Human cytomegalovirus UL23 inhibits transcription of interferon-γ stimulated genes and blocks antiviral interferon-γ responses by interacting with human N-myc interactor protein

**DOI:** 10.1371/journal.ppat.1006867

**Published:** 2018-01-29

**Authors:** Linyuan Feng, Jingxue Sheng, Gia-Phong Vu, Yujun Liu, Chingman Foo, Songbin Wu, Phong Trang, Marco Paliza-Carre, Yanhong Ran, Xiaoping Yang, Xu Sun, Zemin Deng, Tianhong Zhou, Sangwei Lu, Hongjian Li, Fenyong Liu

**Affiliations:** 1 Department of Biotechnology, College of Life Science and Technology, Jinan University, Guangzhou, Guangdong, China; 2 School of Public Health, University of California, Berkeley, Berkeley, California, United States of America; 3 School of Medicine, St. George’s University, Grenada, West Indies; 4 Shandong University of Traditional Chinese Medicine, Jinan, Shandong, China; 5 Keck School of Medicine, University of Southern California, Los Angeles, California, United States of America; University of Wisconsin-Madison, UNITED STATES

## Abstract

Interferon-γ (IFN-γ) represents one of the most important innate immunity responses in a host to combat infections of many human viruses including human herpesviruses. Human N-myc interactor (Nmi) protein, which has been shown to interact with signal transducer and activator of transcription (STAT) proteins including STAT1, is important for the activation of IFN-γ induced STAT1-dependent transcription of many genes responsible for IFN-γ immune responses. However, no proteins encoded by herpesviruses have been reported to interact with Nmi and inhibit Nmi-mediated activation of IFN-γ immune responses to achieve immune evasion from IFN-γ responses. In this study, we show strong evidence that the UL23 protein of human cytomegalovirus (HCMV), a human herpesvirus, specifically interacts with Nmi. This interaction was identified through a yeast two-hybrid screen and co-immunoprecipitation in human cells. We observed that Nmi, when bound to UL23, was not associated with STAT1, suggesting that UL23 binding of Nmi disrupts the interaction of Nmi with STAT1. In cells overexpressing UL23, we observed (a) significantly reduced levels of Nmi and STAT1 in the nuclei, the sites where these proteins act to induce transcription of IFN-γ stimulated genes, and (b) decreased levels of the induction of the transcription of IFN-γ stimulated genes. UL23-deficient HCMV mutants induced higher transcription of IFN-γ stimulated genes and exhibited lower titers than parental and control revertant viruses expressing functional UL23 in IFN-γ treated cells. Thus, UL23 appears to interact directly with Nmi and inhibit nuclear translocation of Nmi and its associated protein STAT1, leading to a decrease of IFN-γ induced responses and an increase of viral resistance to IFN-γ. Our results further highlight the roles of UL23-Nmi interactions in facilitating viral immune escape from IFN-γ responses and enhancing viral resistance to IFN antiviral effects.

## Author summary

Interferon-γ (IFN-γ) responses are vital for a host to combat infections of many human viruses including human herpesviruses. Upon treatment of IFN-γ, transcription of many genes responsible for IFN-γ immune responses is activated primarily by the signal transducer and activator of transcription (STAT) proteins such as STAT1 protein. Human N-myc interactor (Nmi) protein has been shown to interact with STAT proteins including STAT1 and activate IFN-γ induced STAT-dependent transcription. However, no proteins encoded by herpesviruses have been reported to interact with Nmi and inhibit Nmi-mediated activation of IFN-γ immune responses to achieve immune evasion from IFN-γ responses. In this study, we show strong evidence that the UL23 protein of human cytomegalovirus (HCMV), a human herpesvirus, specifically interacts with Nmi protein. UL23 appears to interact directly with Nmi and inhibit nuclear translocation of Nmi and its associated protein STAT1, leading to a decrease of IFN-γ responses and an increase of viral resistance to IFN-γ. Blocking UL23 expression led to higher transcription of IFN-γ stimulated genes and significant inhibition of viral growth in infected cells. These results suggest that interfering with Nmi function may represent an effective mechanism for a herpesvirus to block Nmi-mediated IFN-γ responses and increase viral resistance to IFN-γ. This also provides a potentially new therapeutic strategy to treat HCMV infection by modulating Nmi activity with blocking the expression of a viral protein.

## Introduction

Human cytomegalovirus (CMV), a member of the human herpesvirus family, is a common opportunistic virus causing severe ailments and deaths in people with immature or compromised immune systems [1–4]. HCMV ability to evade the host immune system can significantly impact the course of illness. For example, predisposition to bacterial and fungal infections is common for HCMV positive patients undergoing hematopoietic stem cell transplant procedure [5,6]. HCMV expresses viral proteins to modulate the host immune responses at every step of its life cycle, which play a crucial role in viral pathogenesis [7–9].

Interferons (IFNs) such as IFN-γ are part of the innate immune response to viral infections and confer potent antiviral effects. These cytokines also have important roles in immune-surveillance for malignant cells [10,11]. IFN-γ, a type II IFN, binds to a cell-surface receptor, which is known as the type II IFN receptor [12–14]. Upon viral infection, IFN-γ [15,16] activates cellular signaling networks, such as the JAK–STAT pathway [17,18]. The transcription of type II IFN (IFN-γ)-dependent genes is regulated by gamma-activated sequence (GAS) elements, and the signal transducer and activator of transcription (STAT) protein, STAT1 is the most important transcription factor for the regulation of these transcriptional responses. After JAK1 and JAK2 are activated via the binding of IFN-γ to its receptor, they then regulate the downstream phosphorylation of STAT1 to form STAT1–STAT1 homodimers. The homodimers are transported to the nucleus and bind to GAS elements to induce transcription of various interferon stimulated genes [19–22].

The STAT family of transcription factors regulates many cellular processes including cell growth, apoptosis, immune responses, and oncogenesis [20,23,24]. STATs have been found to interact with various proteins, which help to modulate STAT signaling and mediate crosstalk with other cellular signaling pathways [25,26]. Nmi (N-Myc interactor) is one of these proteins, which was first identified through a yeast two-hybrid screen [27]. Nmi interacts with all STATs except STAT2 [27–29], and has been shown to augment STAT-mediated transcription in response to IL-2 and IFN-γ by recruiting transcription factors CBP/p300 and potentiating their interactions with STAT proteins [30]. Because of the vital role of Nmi in IFN-γ response, it is reasonable to suggest that a virus may encode a protein to modulate Nmi function in order to escape IFN-γ-induced antiviral responses. However, no proteins encoded by any herpesviruses have been reported to modulate Nmi function and diminish IFN-γ responses, leading to increased viral resistance to IFN-γ and escape from IFN-γ responses.

In order to establish persistent and latent infection in healthy individuals, HCMV encodes a large array of proteins that can modulate different components and pathways of the immune responses including MHC antigen presentation and NK cell activation [31–33]. For example, HCMV IE1 protein has been shown to interact with STAT proteins (e.g. STAT2) and elicit a type II IFN-like host cell response that depends on activated STAT1 but not interferon-γ [31,34–37]. In this report, we provide the first direct evidence that HCMV UL23 interacts with Nmi and disrupts its interaction with STAT1. UL23 is a virion protein found in the tegument and is expressed in the cytoplasm in HCMV infected cells [38]. However, UL23 is dispensable for viral replication in cultured cells and little is currently known about its function [39]. Binding of UL23 to Nmi resulted in the cytoplasmic localization of Nmi and STAT1 and led to a decreased level of transcription of the IFN-γ stimulated genes. In IFN-γ treated cells, HCMV mutants lacking UL23 expression induced much higher levels of transcription of IFN-stimulated genes and were much more sensitive to the antiviral effect of IFN-γ than the parental virus with UL23 expression. Our results suggest that UL23 represents the first herpesvirus-encoded protein that interacts with Nmi and inhibits the transcription of IFN-γ stimulated genes, leading to HCMV resistance to the antiviral effect of IFN-γ. Modulating the function of Nmi may represent a novel mechanism for a herpesvirus to escape from IFN-γ response and increase its resistance to IFN-γ.

## Results

### Identification of the interaction of UL23 and Nmi by yeast two-hybrid analysis and coimmunoprecipitation (co-IP)

We used a yeast two-hybrid system to screen for cellular partners that potentially interact with HCMV UL23 by transforming *S*. *cerevisiae* AH109 containing pGBKT7-UL23 with a cDNA library derived from human embryonic kidney. We consistently observed in our yeast two-hybrid screens a positive interaction between pGBKT7-UL23 and pACT2-Nmi, which contained the full-length coding sequence of Nmi (S1 Fig). Of approximately 1 x 10^7^ independent cDNA clones tested, 44 yeast colonies yielded positive results. Of these 44 positive clones, 12 contained plasmid constructs with the full-length and partial coding sequence of Nmi, while each of the remaining 32 clones contained the sequence of a unique human gene (S1 Table). Nmi has been shown to bind to several STAT proteins including STAT1 and induce IFN-γ induced STAT1-dependent transcription by recruiting transcription factors CBP/p300 and potentiating their interactions with STAT proteins [40–42].

**Fig 1:**
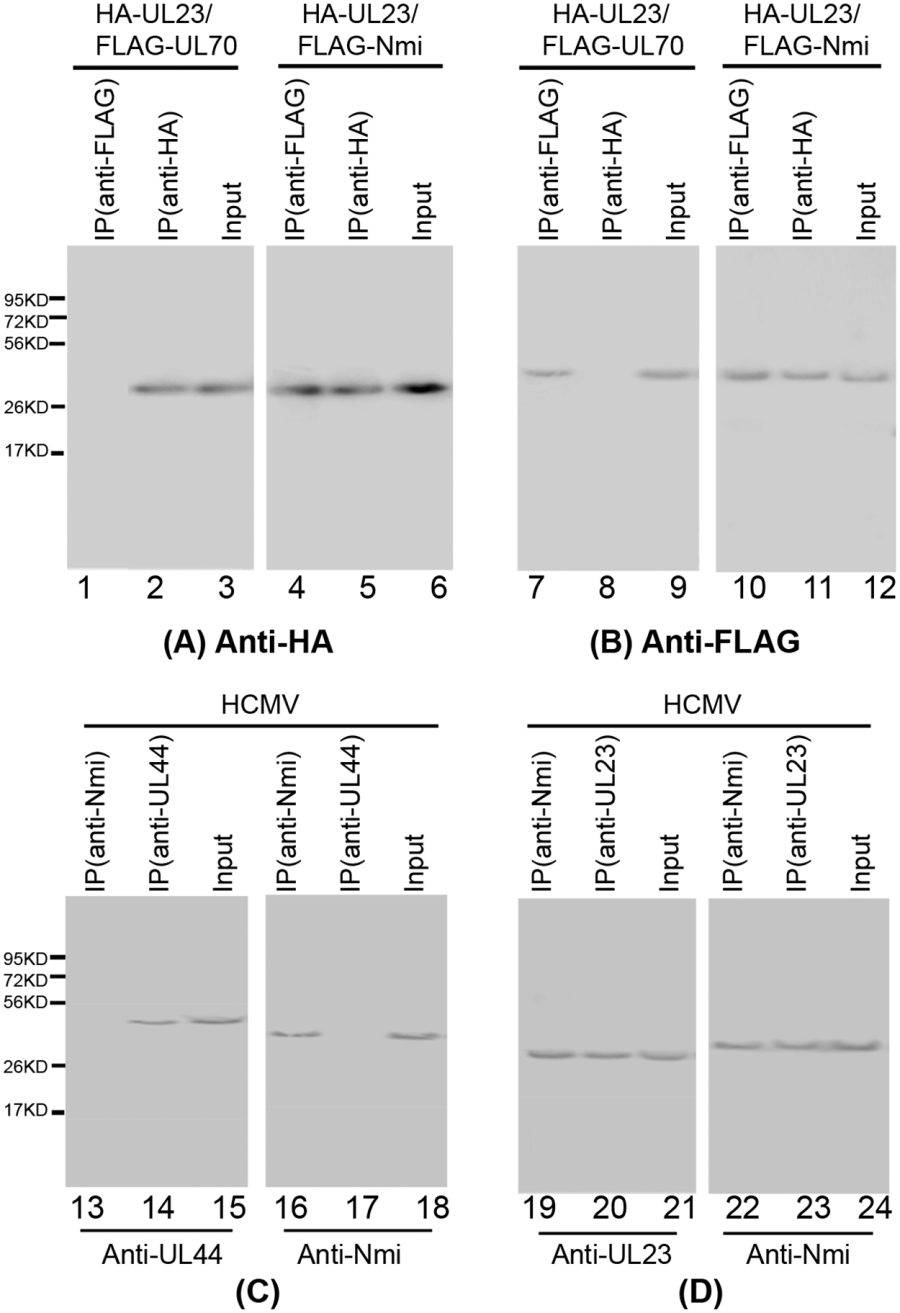
Interaction between UL23 and Nmi identified by coimmunoprecipitation. (A-B) Human U251 cells were co-transfected with a combination of two plasmids expressing FLAG- and HA-tagged proteins, and then harvested at 48 hours posttransfection. (C-D) Human U251 cells were infected with HCMV (MOI = 1) and cellular lysates were prepared at 48–72 hours postinfection. The input protein samples (80 μg) (Input) (lanes 3, 6, 9, 12, 15, 18, 21, and 24) and samples (15 μg) that were precipitated with anti-HA (IP (anti-HA)) (lanes 2, 5, 8, and 11), anti-FLAG (IP (anti-FLAG)) (lanes 1, 4, 7, and 10), anti-Nmi (lanes 13, 16, 19, and 22), anti-UL44 (lanes 14 and 17), or anti-UL23 antibodies (lanes 20 and 23), were separated on SDS-containing polyacrylamide gels, and assayed with Western blot analysis using anti-HA (anti-HA) (A), anti-FLAG (anti-FLAG) (B), anti-UL44 (anti-UL44) (C), anti-UL23 (anti-UL23) (D), and anti-Nmi (anti-Nmi) (C-D) antibodies that were directly conjugated to alkaline phosphatase (Abcam, Cambridge, MA), respectively.

Co-IP experiments were performed to determine whether the interaction between UL23 and Nmi occurs in human cells. In these experiments, UL23 HA and Nmi FLAG epitope tag proteins were generated by constructing pCMV-HA-UL23 and pCMV-FLAG-Nmi mammalian expression vectors. The mammalian expression constructs were transfected into human U251 cells and proteins were extracted at 48 hours. Protein samples were separated by SDS-PAGE, transferred to membranes, and probed with anti-HA and anti-FLAG antibodies. HCMV UL23 and Nmi were detected as proteins of about 33 and 38 kDa, respectively (Fig 1A and 1B), consistent with their coding sequences of 284 and 307 amino acids, as predicted from the HCMV Towne_BAC_ and human genomic sequences [39,43].

The co-IP experiments with HA-tagged UL23 and the FLAG-tagged Nmi proteins in U251 cells confirmed that UL23 was associated with Nmi (Fig 1A and 1B). Protein lysates from the transfected cells were first either immunoprecipitated with anti-HA or anti-FLAG antibodies, and immunoblotted with antibodies against the HA and FLAG epitope tags. The HA-tagged UL23 was co-precipitated with the FLAG-tagged Nmi (Fig 1A and 1B). In contrast, when using the FLAG-tagged UL70 protein of HCMV that is not known to interact with UL23 as negative control, we observed no significant binding or co-precipitation with the HA-tagged UL23 (Fig 1A and 1B, lanes 1–3 and 7–9) [44]. These results confirmed the specificity of the UL23-Nmi interaction in the co-IP assay and suggested that it may occur in human cells. Similar interaction between HA-tagged UL23 and FLAG-tagged Nmi proteins were also observed in HeLa cells and HCMV-infected U251 cells.

It is possible that UL23 or Nmi may bind non-specifically to tagged or over expressed proteins. Meanwhile, it is important to study if native (untagged UL23) can interact with endogenous Nmi in human cells during HCMV infection. To determine if this is the case, we used UL23 proteins expressed in *E*. *coli* as the antigen to generate an anti-UL23 monoclonal antibody in collaboration with Promab, Inc (Albany, CA). We screened a large number of anti-UL23 antibody-producing clones and selected the clones that generated anti-UL23 antibodies with excellent reactivity and specificity. To determine whether Nmi also interacts with other viral proteins in addition to UL23, we investigated if Nmi is associated with the HCMV polymerase processivity factor, UL44 protein, which is essential for viral DNA replication [4]. Protein lysates from HCMV-infected U251 cells were first either immunoprecipitated with anti-UL23, anti-UL44, or anti-Nmi antibodies, and then immunoblotted with antibodies against UL23, UL44, and Nmi. UL23 was found to be co-precipitated with the endogenous Nmi (Fig 1C and 1D, lanes 19 and 23) while we observed no significant binding or co-precipitation between UL44 and Nmi (Fig 1C and 1D, lanes 13–18). These results suggest that Nmi may specifically interact with UL23 but not UL44 during HCMV infection.

### Delineation of the regions of UL23 and Nmi mediating their interaction

**Fig 2:**
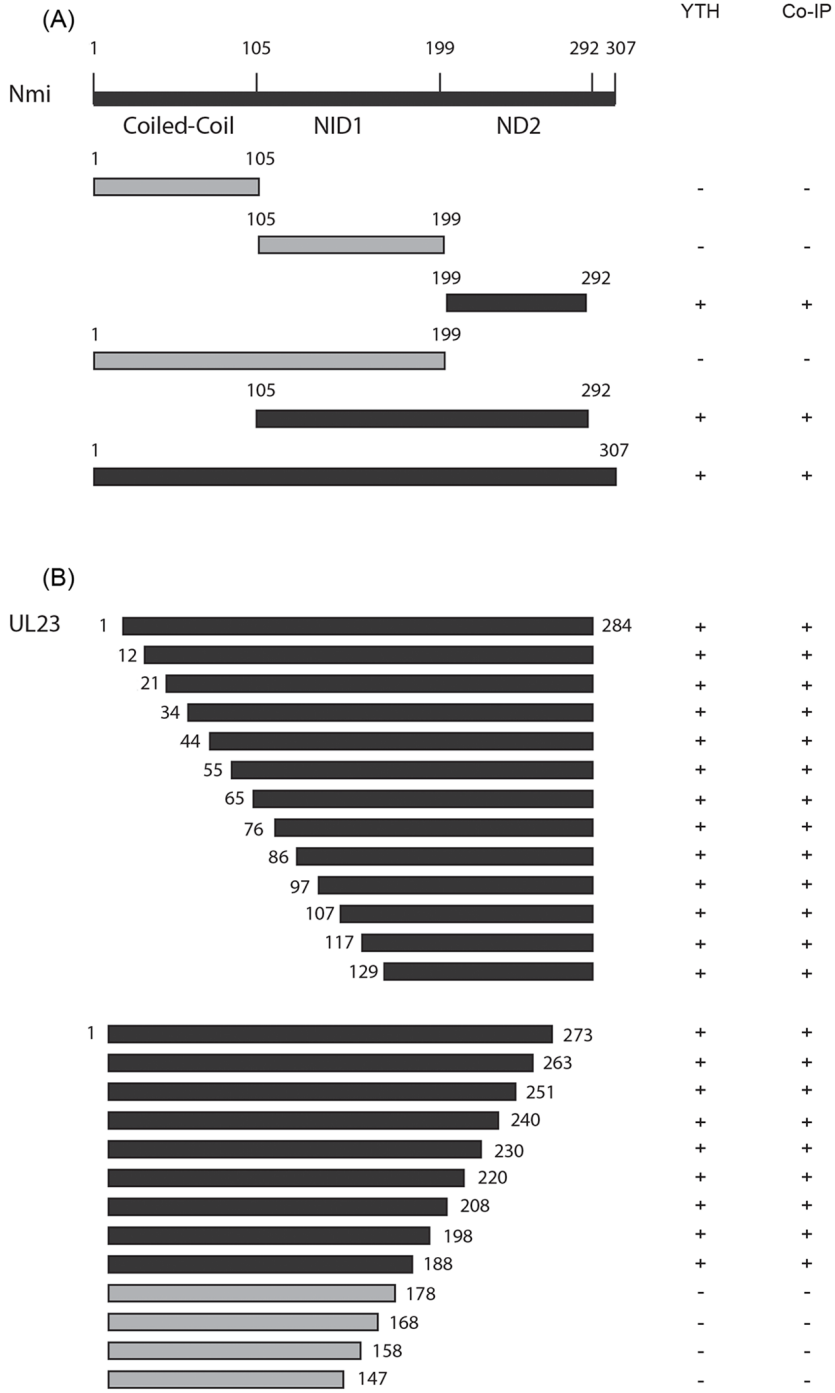
Schematic diagram of Nmi (A) and UL23 (B) and their deletion mutants that interact with each other as identified by the two hybrid screen in yeast (YTH) and co-immunoprecipitation (co-IP) in U251 cells. The interactions that were positive and negative in the two hybrid screen or co-IP were marked as “+” and “-”, respectively.

As a protein known to interact with STAT proteins, Nmi has three distinct domains: (i) a highly conserved coiled-coil domain at the amino terminus (amino acid 1–105); (ii) a NID1 domain (amino acid 105–199); and (iii) a NID2 domain (amino acid 199–307), with its COOH-terminal region showing homology to interferon-induced leucine zipper protein IP35 (Fig 2) [27,45,46]. The STAT binding domains of Nmi are in amino acids 57–99 and 143–202 [30]. To further map the domains of association between UL23 and Nmi, a series of truncation mutants of UL23 and Nmi were constructed (Fig 2) and yeast two-hybrid analyses were carried out to investigate the binary interactions of mutants and full-length proteins (S1 Fig). We also constructed a series of UL23 and FLAG-tagged Nmi truncation mutants (Fig 2, Table 1). Co-IP experiments were carried out to examine the interactions between UL23 and different FLAG-tagged Nmi mutants after the FLAG tagged constructs were transfected into U251 cells. The results, summarized in Fig 2A, indicate that the minimal Nmi mutant that binds to UL23 contains amino acid 199 to 292 and covers the NID2 domain. These results suggest that amino acids 199–292 of Nmi bearing the NID2 domain, a critical domain for Nmi homo-and hetero-dimerization [40,47], is necessary for its association with UL23. Similarly, experiments were performed to study the association of Nmi and a series of truncated FLAG-tagged UL23 (Fig 2B, S1 Fig). The results showed that the minimal UL23 mutant containing amino acids 129 to 284 was capable of binding to Nmi and mutant with carboxyl-terminal deletion from amino acid 179 to 284 failed to bind to UL23 (Fig 2B). Thus, the carboxyl-terminal sequence of UL23 is essential for its interaction with Nmi.

**Table 1:** Plasmid constructs used in the study.

Plasmids	Description	Reference/source
pGBKT7	Vector for protein expression fused with GAL4 DNA-binding domain in yeast	Clontech
pGADT7	Vector for protein expression fused with GAL4 activation domain in yeast	Clontech
pCDNA3.1(+)	Cloning vector for protein expression without tag in mammalian cell	Clontech
pGADT7-Nmi	pGADT7 containing human Nmi full-length sequence (1–924)	This study
pGBKT7-UL23	pGBKT7 containing HCMV UL23 full-length sequence (1–2841)	This study
pCMV-HA	Cloning vector for protein expression fused with HA tag in mammalian cell	Invitrogen
pCMV-FLAG	Cloning vector for protein expression fused with the FLAG tag in mammalian cell	Invitrogen
pCMV-HA-UL23	pCMV-HA containing HCMV UL23 full-length sequence (1–2841) fused with the HA tag	This study
pCMV-FLAG-UL23	pCMV-FLAG containing HCMV UL23 full-length sequence (1–2841) fused with the FLAG tag	This study
pCMV-FLAG-Nmi	pCMV-FLAG containing human Nmi full-length sequence (1–924) fused with the FLAG tag	This study
pCDNA-UL23-HA	pCDNA containing HCMV UL23 full-length sequence (1–2841) fused with a HA tag	This study
pCDNA-UL23-FLAG	pCDNA containing HCMV UL23 full-length sequence (1–2841) fused with the FLAG tag	This study
pCDNA-UL23	pCDNA containing HCMV UL23 full-length sequence (1–2841)	This study
pCDNA-Nmi	pCDNA containing human Nmi full-length sequence (1–2841)	This study
pEGFP-N1	Cloning vector for protein expression fused with EGFP	Invitrogen
pEGFP-UL23	pEGFP containing HCMV UL23 full-length sequence (1–2841)	This study
pDsRed2-	Cloning vector for protein expression fused with DsRed2	Invitrogen
pDsRed2-Nmi	pDsRed2 containing human Nmi full-length sequence (1–2841)	This study
pGL3-Promoter	Cloning vector for luciferase activity fused with pGL3-Promoter	Promega
pGL3-Promoter-3×GAS	pGL3-Promoter containing 3×IFN-γ activating sequence (48bp)	This study
pGADT7-Nmi(1-307aa)	pGADT7 containing human Nmi full-length sequence (1–924)	This study
pGADT7-Nmi(1-105aa)	pGADT7 containing human Nmi C-terminally truncated sequence (1–315)	This study
pGADT7-Nmi(103-199aa)	pGADT7 containing human Nmi N-terminally truncated sequence (307–597)	This study
pGADT7-Nmi(199-292aa)	pGADT7 containing human Nmi N-terminally truncated sequence (595–876)	This study
pGADT7-Nmi(1-199aa)	pGADT7 containing human Nmi C-terminally truncated sequence (1–597)	This study
pGADT7-Nmi(103-292aa)	pGADT7 containing human Nmi N-terminally truncated sequence (307–876)	This study
pCMV-FLAG-Nmi(1-307aa)	pCMV-FLAG containing human Nmi full-length sequence (1–924)	This study
pCMV-FLAG-Nmi(1-105aa)	pCMV-FLAG containing human Nmi C-terminally truncated sequence (1–315)	This study
pCMV-FLAG-Nmi(103-199aa)	pCMV-FLAG containing human Nmi N-terminally truncated sequence (307–597)	This study
pCMV-FLAG-Nmi(199-292aa)	pCMV-FLAG containing human Nmi N-terminally truncated sequence (595–876)	This study
pCMV-FLAG-Nmi(1-199aa)	pCMV-FLAG containing human Nmi C-terminally truncated sequence (1–597)	This study
pCMV-FLAG-Nmi(103-292aa)	pCMV-FLAG containing human Nmi N-terminally truncated sequence (307–876)	This study
pGBKT7-UL23(1-284aa)	pGBKT7 containing HCMV UL23 C-terminally truncated sequence (1–855)	This study
pGBKT7-UL23(12-284aa)	pGBKT7 containing HCMV UL23 N-terminally truncated sequence (34–855)	This study
pGBKT7-UL23(21-284aa)	pGBKT7 containing HCMV UL23 N-terminally truncated sequence (1–855)	This study
pGBKT7-UL23(34-284aa)	pGBKT7 containing HCMV UL23 N-terminally truncated sequence (100–855)	This study
pGBKT7-UL23(44-284aa)	pGBKT7 containing HCMV UL23 N-terminally truncated sequence (130–855)	This study
pGBKT7-UL23(55-284aa)	pGBKT7 containing HCMV UL23 N-terminally truncated sequence (163–855)	This study
pGBKT7-UL23(65-284aa)	pGBKT7 containing HCMV UL23 N-terminally truncated sequence (193–855)	This study
pGBKT7-UL23(76-284aa)	pGBKT7 containing HCMV UL23 N-terminally truncated sequence (226–855)	This study
pGBKT7-UL23(86-284aa)	pGBKT7 containing HCMV UL23 N-terminally truncated sequence (256–855)	This study
pGBKT7-UL23(97-284aa)	pGBKT7 containing HCMV UL23 N-terminally truncated sequence (289–855)	This study
pGBKT7-UL23(107-284aa)	pGBKT7 containing HCMV UL23 N-terminally truncated sequence (319–411)	This study
pGBKT7-UL23(117-284aa)	pGBKT7 containing HCMV UL23 N-terminally truncated sequence (349–855)	This study
pGBKT7-UL23(129-284aa)	pGBKT7 containing HCMV UL23 N-terminally truncated sequence (385–855)	This study
pGBKT7-UL23(1-273aa)	pGBKT7 containing HCMV UL23 C-terminally truncated sequence (1–819)	This study
pGBKT7-UL23(1-263aa)	pGBKT7 containing HCMV UL23 C-terminally truncated sequence (1–789)	This study
pGBKT7-UL23(1-251aa)	pGBKT7 containing HCMV UL23 C-terminally truncated sequence (1–753)	This study
pGBKT7-UL23(1-240aa)	pGBKT7 containing HCMV UL23 C-terminally truncated sequence (1–720)	This study
pGBKT7-UL23(1-230aa)	pGBKT7 containing HCMV UL23 C-terminally truncated sequence (1–690)	This study
pGBKT7-UL23(1-220aa)	pGBKT7 containing HCMV UL23 C-terminally truncated sequence (1–660)	This study
pGBKT7-UL23(1-208aa)	pGBKT7 containing HCMV UL23 C-terminally truncated sequence (1–624)	This study
pGBKT7-UL23(1-198aa)	pGBKT7 containing HCMV UL23 C-terminally truncated sequence (1–594)	This study
pGBKT7-UL23(1-188aa)	pGBKT7 containing HCMV UL23 C-terminally truncated sequence (1–564)	This study
pGBKT7-UL23(1-178aa)	pGBKT7 containing HCMV UL23 C-terminally truncated sequence (1–534)	This study
pGBKT7-UL23(1-168aa)	pGBKT7 containing HCMV UL23 C-terminally truncated sequence (1–504)	This study
pGBKT7-UL23(1-158aa)	pGBKT7 containing HCMV UL23 C-terminally truncated sequence (1–474)	This study
pGBKT7-UL23(1-147aa)	pGBKT7 containing HCMV UL23 C-terminally truncated sequence (1–441)	This study
pCMV-FLAG-UL23(1-284aa)	pCMV-FLAG containing HCMV UL23 C-terminally truncated sequence (1–855)	This study
pCMV-FLAG-UL23(12-284aa)	pCMV-FLAG containing HCMV UL23 N-terminally truncated sequence (34–855)	This study
pCMV-FLAG-UL23(21-284aa)	pCMV-FLAG containing HCMV UL23 N-terminally truncated sequence (1–855)	This study
pCMV-FLAG-UL23(34-284aa)	pCMV-FLAG containing HCMV UL23 N-terminally truncated sequence (100–855)	This study
pCMV-FLAG-UL23(44-284aa)	pCMV-FLAG containing HCMV UL23 N-terminally truncated sequence (130–855)	This study
pCMV-FLAG-UL23(55-284aa)	pCMV-FLAG containing HCMV UL23 N-terminally truncated sequence (163–855)	This study
pCMV-FLAG-UL23(65-284aa)	pCMV-FLAG containing HCMV UL23 N-terminally truncated sequence (193–855)	This study
pCMV-FLAG-UL23(76-284aa)	pCMV-FLAG containing HCMV UL23 N-terminally truncated sequence (226–855)	This study
pCMV-FLAG-UL23(86-284aa)	pCMV-FLAG containing HCMV UL23 N-terminally truncated sequence (256–855)	This study
pCMV-FLAG-UL23(97-284aa)	pCMV-FLAG containing HCMV UL23 N-terminally truncated sequence (289–855)	This study
pCMV-FLAG-UL23(107-284aa)	pCMV-FLAG containing HCMV UL23 N-terminally truncated sequence (319–411)	This study
pCMV-FLAG-UL23(117-284aa)	pCMV-FLAG containing HCMV UL23 N-terminally truncated sequence (349–855)	This study
pCMV-FLAG-UL23(129-284aa)	pCMV-FLAG containing HCMV UL23 N-terminally truncated sequence (385–855)	This study
pCMV-FLAG-UL23(1-273aa)	pCMV-FLAG containing HCMV UL23 C-terminally truncated sequence (1–819)	This study
pCMV-FLAG-UL23(1-263aa)	pCMV-FLAG containing HCMV UL23 C-terminally truncated sequence (1–789)	This study
pCMV-FLAG-UL23(1-251aa)	pCMV-FLAG containing HCMV UL23 C-terminally truncated sequence (1–753)	This study
pCMV-FLAG-UL23(1-240aa)	pCMV-FLAG containing HCMV UL23 C-terminally truncated sequence (1–720)	This study
pCMV-FLAG-UL23(1-230aa)	pCMV-FLAG containing HCMV UL23 C-terminally truncated sequence (1–690)	This study
pCMV-FLAG-UL23(1-220aa)	pCMV-FLAG containing HCMV UL23 C-terminally truncated sequence (1–660)	This study
pCMV-FLAG-UL23(1-208aa)	pCMV-FLAG containing HCMV UL23 C-terminally truncated sequence (1–624)	This study
pCMV-FLAG-UL23(1-198aa)	pCMV-FLAG containing HCMV UL23 C-terminally truncated sequence (1–594)	This study
pCMV-FLAG-UL23(1-188aa)	pCMV-FLAG containing HCMV UL23 C-terminally truncated sequence (1–564)	This study
pCMV-FLAG-UL23(1-178aa)	pCMV-FLAG containing HCMV UL23 C-terminally truncated sequence (1–534)	This study
pCMV-FLAG-UL23(1-168aa)	pCMV-FLAG containing HCMV UL23 C-terminally truncated sequence (1–504)	This study
pCMV-FLAG-UL23(1-158aa)	pCMV-FLAG containing HCMV UL23 C-terminally truncated sequence (1–474)	This study
pCMV-FLAG-UL23(1-147aa)	pCMV-FLAG containing HCMV UL23 C-terminally truncated sequence (1–441)	This study

### Blocking of STAT1 interaction with Nmi upon binding to UL23

**Fig 3:**
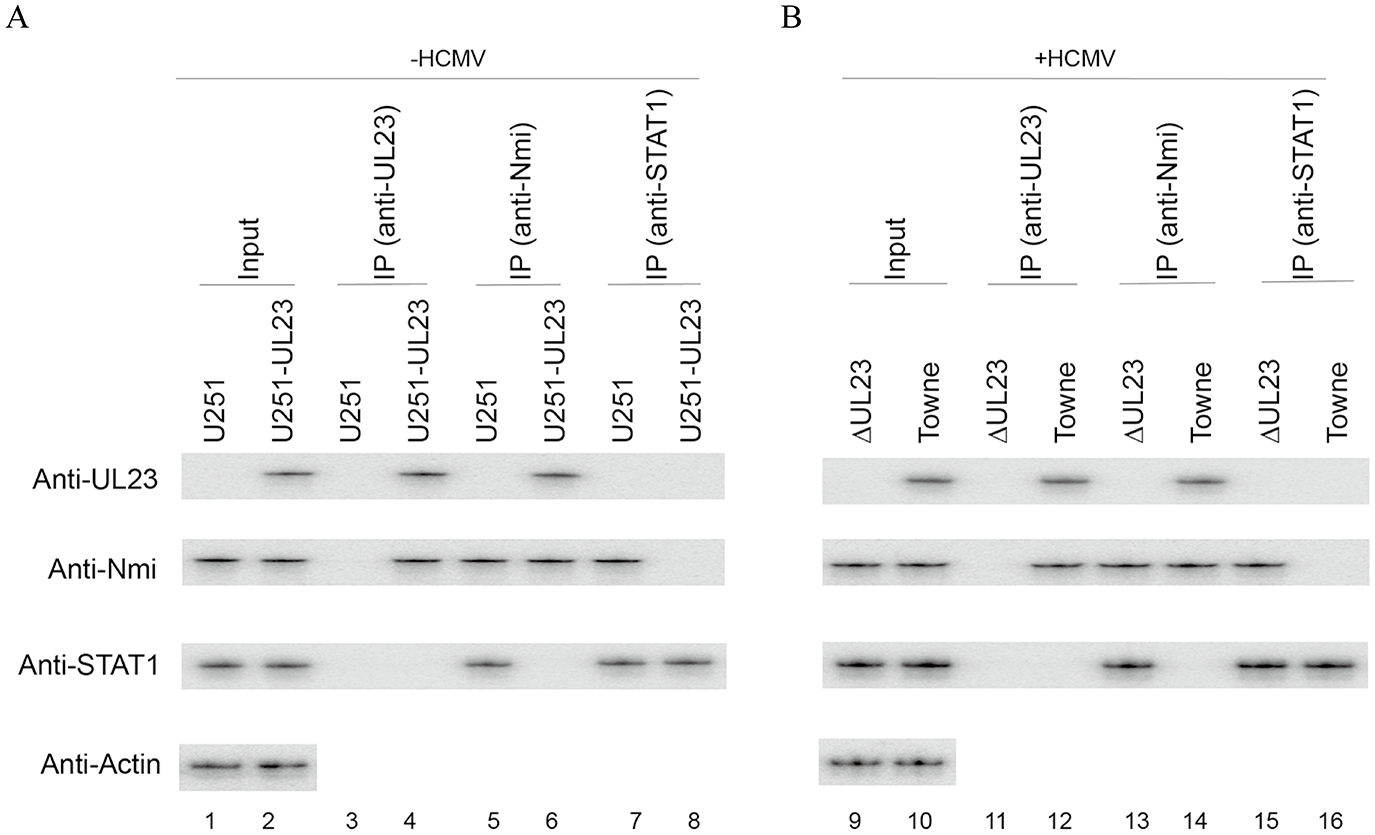
Inhibition of Nmi interaction with STAT1 by UL23 in the absence (lanes 1–8) and presence of HCMV infection (lanes 9–16). (A) U251 (lanes 1, 3, 5, 7) and U251-UL23 cells (lanes 2, 4, 6, 8) were treated with IFN-γ (1000 U/ml) for 12 hours and then harvested to generate protein lysates. (B) The IFN-γ treated U251 cells were infected with HCMV Towne_BAC_ (lanes 10, 12, 14, 16) or ΔUL23 (lanes 9, 11, 13, 15) (MOI = 1) at 12 hours post-treatment and protein lysates were generated at 24–48 hours postinfection. The input protein samples (80 μg) (Input) (lanes 1, 2, 9, and 10) and samples (15 μg) that were precipitated with anti-UL23 (IP (anti-UL23)) (lanes 3, 4, 11, and 12), anti-Nmi (IP (anti-Nmi)) (lanes 5, 6, 13, and 14), or anti-STAT1 (lanes 7, 8, 15, and 16), were separated on SDS-containing polyacrylamide gels and assayed with Western blot analysis with indicated antibodies.

To determine if the binding of UL23 affects the interaction of Nmi with STAT1 protein, cell lines U251-FLAG and U251-FLAG-UL23 that contained empty vector pCMV-FLAG and pCMV-FLAG-UL23, respectively, were constructed, and the expression of FLAG-tagged UL23 protein was confirmed in U251-FLAG-UL23 cells. Cell lines U251-C and U251-UL23 that contained empty control vector pCDNA and construct pCDNA-UL23, respectively, were also constructed from U251 cells, and the expression of UL23 in U251-UL23 cells was confirmed (Fig 3, lane 2). The interactions of Nmi with UL23 and STAT1 were investigated by co-immunoprecipitation experiments with anti-UL23, anti-STAT1, and anti-Nmi antibodies. STAT1 appeared to be associated with Nmi in U251 and U251-C cells but not in U251-UL23 cells when cells were treated with IFN-γ (Fig 3, compare lanes 5 and 6, 7 and 8). Furthermore, Nmi was in a complex containing either UL23 or STAT1 but not in a complex containing both UL23 and STAT1 (Fig 3, lanes 3–8). Similar results were also observed in cells containing empty control vector pCMV-FLAG and construct pCMV-FLAG-UL23. These results suggest that binding of UL23 inhibits the ability of Nmi to interact with STAT1 protein.

To further investigate this issue in the context of HCMV infection, we constructed two mutants, ΔUL23 and UL23stop, which were derived from Towne_BAC_ by deleting the entire ORF sequence of UL23 and introducing a stop codon immediately downstream from its translation initiation codon, respectively. Furthermore, rescued viral mutants, R-ΔUL23 and R-stop, were generated from ΔUL23 and UL23stop, by restoring the UL23 expression, respectively, following the procedures as described previously [39]. U251 cells were treated with IFN-γ and then infected with these viruses. Coimmunoprecipitation experiments confirmed the interactions of Nmi with UL23 in Towne_BAC_ infected cells treated with IFN-γ (Fig 3, lanes 12 and 14). Our results also showed that STAT1 was associated with Nmi in cells infected with ΔUL23 and UL23stop mutants but not with Towne_BAC_ and rescued viruses R-ΔUL23 and R-stop (Fig 3, compared lanes 13 and 14, 15 and 16). Similar results were also observed in IFN-γ treated human foreskin fibroblasts infected with these viruses. These results suggest that binding to Nmi by UL23 abolishes the association of Nmi with STAT1.

### Effect of UL23 on the cellular localization of Nmi and STAT1

**Fig 4:**
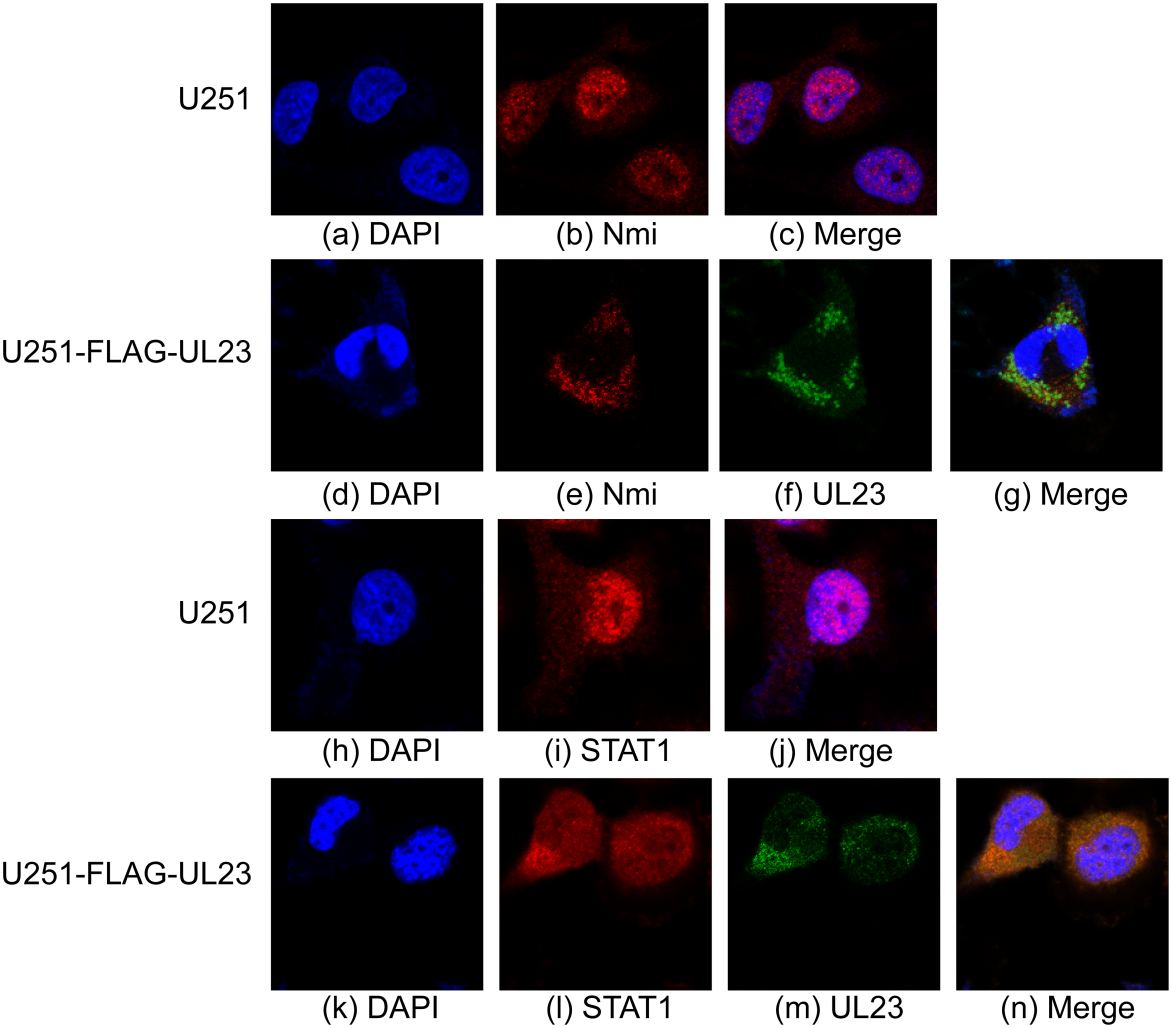
Effect of the expression of UL23 on the cellular distribution of Nmi and STAT1. Immunofluorescence microscopy of the cellular localization of Nmi and STAT1 in the parental U251 cells (U251) and cells overexpressing FLAG-tagged UL23 (U251-FLAG-UL23). Cells were treated with IFN-γ (1000 U/ml), fixed at 48 hours post-treatment, stained with antibodies, and visualized. The images of Nmi (red) (b and e) and STAT1 (red) (i and l) were used to merge with the nuclei stained with DAPI (blue) (a, d, h, k) to generate the composite images (c, g, j, n), in the absence and presence of those of FLAG-tagged UL23 (green) (f and m). The images show different levels of magnification.

UL23 and Nmi are expected to co-localize if they are associated with each other in the cell. UL23, a tegument protein, has been shown to be localized in the cytoplasm [38]. Under certain conditions (e.g. under cellular stress and IFN treatment), Nmi, which binds to multiple STAT proteins and facilitates interactions of these STAT proteins with nuclear transcription factors such as CBP/p300 [48], is localized in the nuclei [25,30,49]. To determine whether UL23 is co-localized with Nmi, the cellular localization of these expressed proteins in U251 and U251-FLAG-UL23 cells treated with IFN-γ was studied using immunofluorescence microscopy. Consistent with previous observations of Nmi as a nuclear protein [25,30,49], the endogenous Nmi protein was found to localize primarily in the nuclei in U251 cells or U251-FLAG cells (Fig 4, S2 Table). In contrast, FLAG-tagged UL23 and Nmi were primarily localized in the cytoplasm in U251-FLAG-UL23 cells (Fig 4). No obvious fluorescence was observed in control cells not treated with anti-Nmi or anti-FLAG antibody, indicating that the staining observed is specific and not due to secondary antibody binding to viral or cellular proteins. Furthermore, these results suggest that the cellular distribution of Nmi is affected by the presence of UL23, which is primarily localized in the cytoplasm.

It is reasonable to suggest that the cellular distribution of STAT1 protein is affected by UL23 because UL23 affects the cellular localization of Nmi, a STAT1 binding protein. To determine if this is the case, cell lines U251 and U251-FLAG-UL23 were treated with IFN-γ and studied using immunofluorescence microscopy. STAT1 and Nmi appeared to be predominantly in the nuclei of U251 and U251-FLAG cells but were found to be primarily localized in the cytoplasm of U251-FLAG-UL23 cells that expressed FLAG-UL23 (Fig 4, S2 Table). These observations indicate that UL23 expression affects the cellular localization of Nmi protein as well as STAT1 protein.

To confirm these results and investigate cellular localization of untagged UL23, Nmi, and STAT1 proteins, cell lines U251-C and U251-UL23, which contained empty vector pCDNA and pCDNA-UL23 respectively, were treated with IFN-γ and stained with antibodies. Similar to the results with the tagged proteins (Fig 4), Nmi and STAT1 proteins were found primarily in the nuclei of U251-C cells (S2 Table). In contrast, these proteins, along with UL23, were found to be primarily expressed in the cytoplasm of U251-UL23 cells (S2 Table). Combined with the results using tagged-UL23 constructs, these results indicate that the FLAG tag sequence in FLAG-UL23 does not affect the interaction and co-localization of UL23 with Nmi, and that UL23 specifically interacts with and affects the cellular distribution of Nmi. Furthermore, our results suggest that UL23 may bind and retain Nmi to the cytoplasm and affect the interaction of Nmi with STAT1, leading to the modulation of the cellular distribution of STAT1 in the cytoplasm.

**Fig 5:**
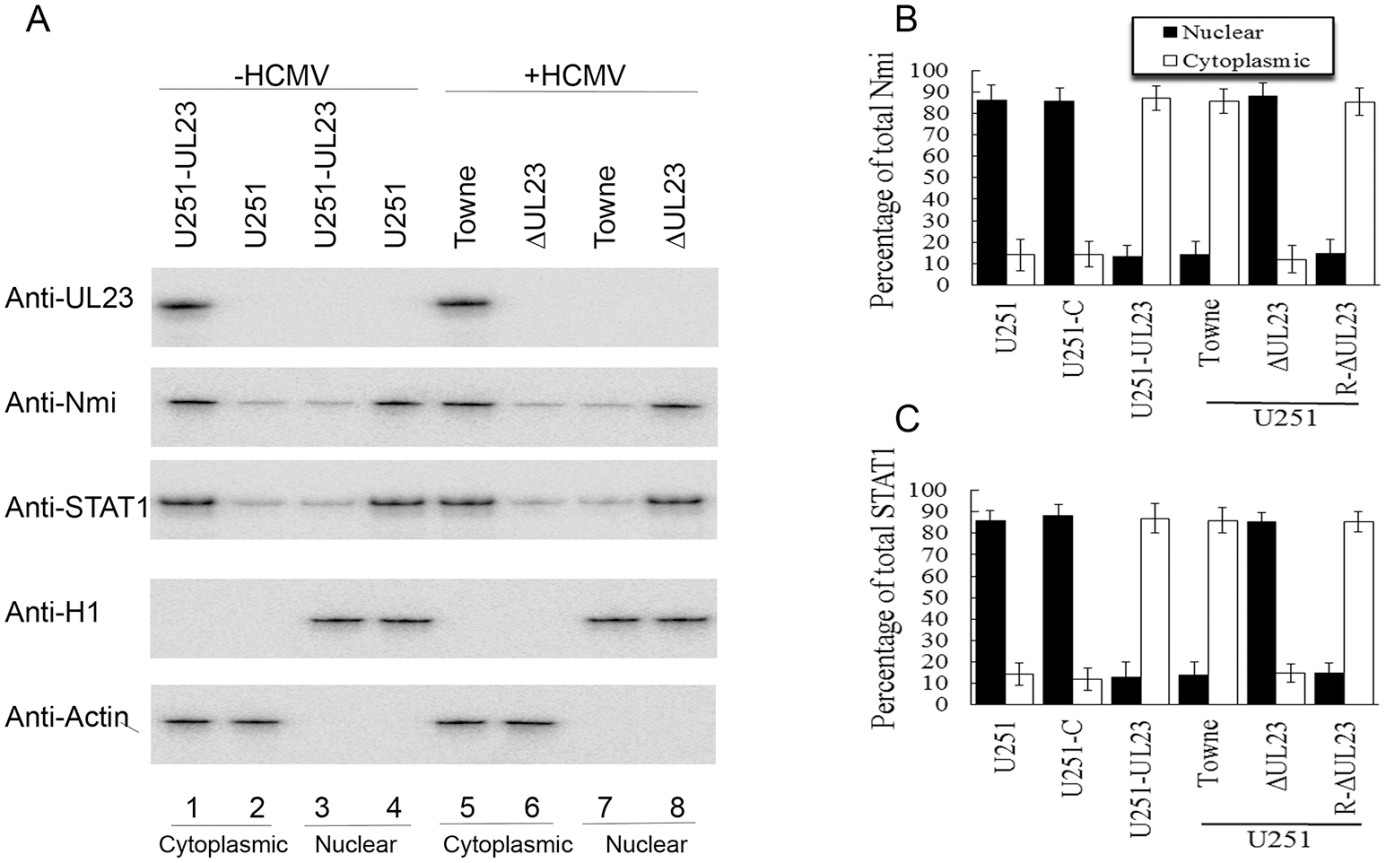
Effect of the expression of UL23 on the distribution of Nmi and STAT1 in nuclear and cytoplasmic fractions. (A) U251 (lanes 2 and 4) and U251-UL23 cells (lanes 1 and 3) were treated with IFN-γ (1000 U/ml) and harvested at 36 hours post-treatment. The IFN-γ treated U251 cells were infected with HCMV Towne_BAC_ (lanes 5 and 7) or ΔUL23 (lanes 6 and 8) (MOI = 1) at 12 hours post-treatment and harvested at 24 hours postinfection. The harvested cells were separated into nuclear and cytoplasmic fractions. Equivalent amounts of each fraction were analyzed by immunoblotting with anti-UL23, anti-Nmi, and anti-STAT1. The purity of the nuclear and cytoplasmic fractions was assayed by immunoblotting with anti-histone H1 and anti-actin, respectively. The membranes were reacted with antibodies and quantitated with a STORM840 PhosphorImager (GE Healthcare) or a Gel Documentation Station (BioRad, Hercules, CA) [60,64]. The protein levels of Nmi (B) and STAT1 (C) in the nuclei and cytoplasm of different cells that were mock-infected or infected with different viruses were quantified. The experiments were repeated three times. The standard deviation is indicated by the error bar.

To further determine the effects of UL23 expression on the localization of Nmi and STAT1, we performed cell fractionation and Western blot analysis to look at the distribution of these proteins (Fig 5). Different cells (e.g. U251, U251-C, and U251-UL23 cells) were treated with IFN-γ and then harvested. Furthermore, in order to study the effects of UL23 expression on the localization of Nmi and STAT1 in the context of HCMV infection, U251 cells were treated with IFN-γ, infected with Towne_BAC_, ΔUL23, or R-ΔUL23, and the cell extracts were separated into nuclear and cytoplasmic fractions by centrifugation. The purity of the fractions was confirmed by immunoblotting for histone H1 (nuclear marker) and actin (cytoplasmic marker) (Fig 5A). In uninfected parental U251 cells, more than 80% of Nmi and STAT1 was found in the nuclei while less than 20% of these proteins was in the cytoplasm (Fig 5A, lanes 2 and 4). Similar results were also found in control U251-C cells that contained the empty expression vector (Fig 5B and 5C). In contrast, less than 20% of these two proteins (i.e. Nmi and STAT1) along with UL23 was found in the nuclear fractions while more than 80% of these three proteins was in cytoplasmic fractions in uninfected U251-UL23 cells (Fig 5A, lanes 1 and 3, Fig 5B and 5C). In U251 cells infected with mutant ΔUL23, more than 80% of Nmi and STAT1 was found in the nuclei while less than 20% of these proteins was in the cytoplasm (Fig 5B, lanes 6 and 8, Fig 5B and 5C). In contrast, less than 20% of Nmi and STAT1 along with UL23 was found in the nuclear fractions while more than 80% of these three proteins was in cytoplasmic fractions in U251 cells infected with parental virus Towne_BAC_ and rescued virus R-ΔUL23 (Fig 5A, lanes 5 and 7, Fig 5B and 5C). These findings validate our immunofluorescence microscopy experiments results (Fig 4, S2 Table) and suggest that UL23 plays an important role in the cellular localization of Nmi and STAT1.

### Effect of UL23 on the induction of transcription of IFN-γ-stimulated genes mediated by the interaction between Nmi and STAT1

STAT1 protein is among the most important transcription factors responsible for the induction of transcription of IFN-γ stimulated genes [20,23,24]. To activate the transcription of IFN-γ stimulated genes, STAT1 needs to be localized in the nuclei and recognizes the gamma-activated sites (GAS) of these genes [30]. If UL23 interaction of Nmi leads to the cytoplasmic accumulation of STAT1, it is expected that expression of UL23 may inhibit the STAT1-dependent transcription of IFN-γ stimulated genes. Three sets of experiments were carried out to determine if this is the case.

**Fig 6:**
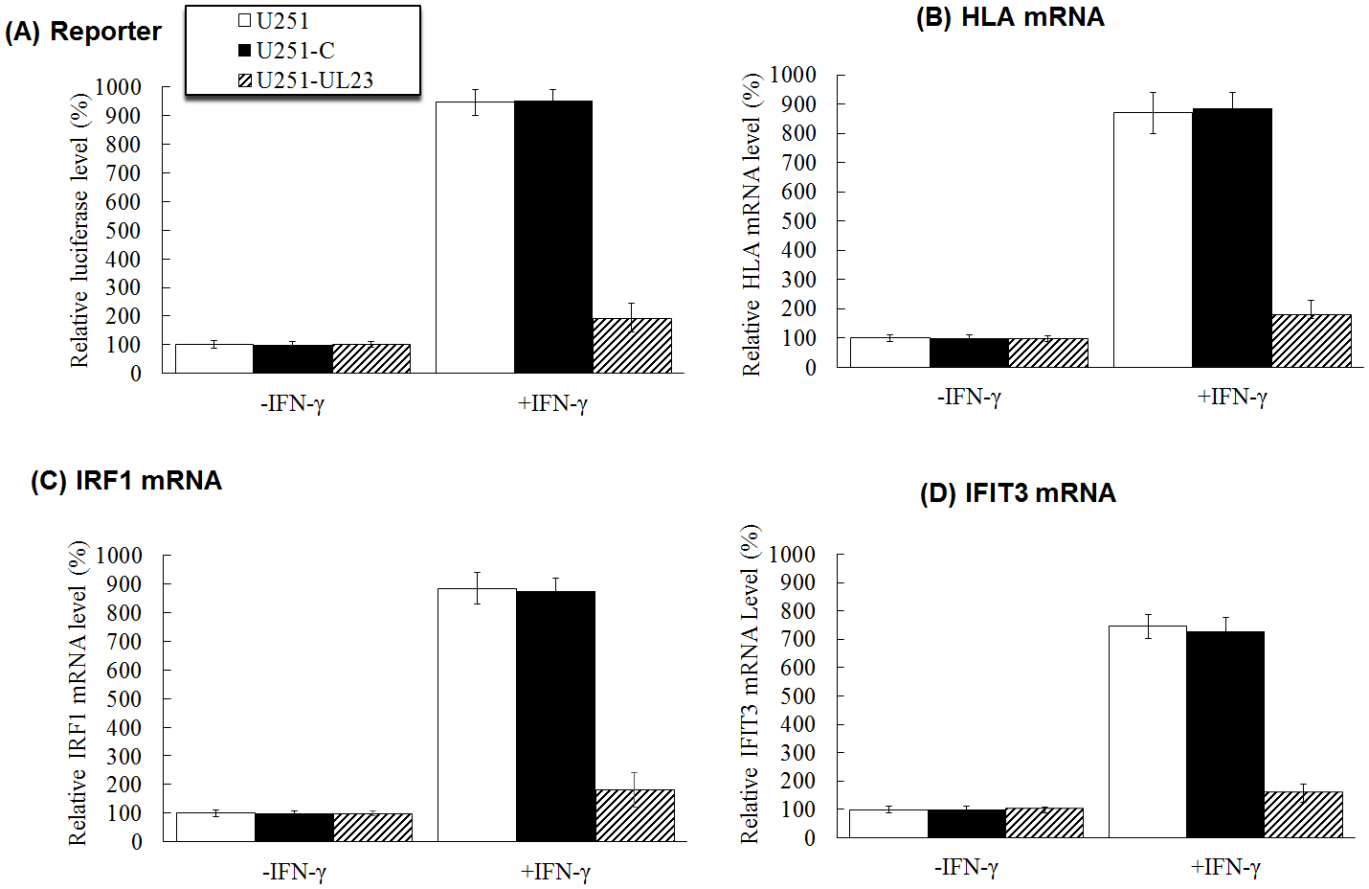
Inhibition of IFN-γ induced transcription by UL23. (A) U251, U251-C, and U251-UL23 cells were transfected with reporter plasmid pGL3-Promoter-3×GAS and the internal control reporter pRL-TK. After 24 hours, cells were cultured in the absence (-IFN-γ) and presence (+IFN-γ) of IFN-γ (1000 U/ml) for 24 hours. Luciferase activity was determined luminometrically as relative light units (RLU). (B-D) U251, U251-C, and U251-UL23 cells were cultured in the absence (-IFN-γ) and presence (+IFN-γ) of IFN-γ (1000 U/ml). After 24 hours, total RNAs were extracted from cells. The levels of the *HLA-B* (B), *IRF1* (C), and *IFIT3* mRNAs (D) were determined by quantitative reverse transcription PCR (qRT-PCR) using those of actin as the internal control. The values of the relative luciferase and mRNA level represent the ratios of the levels of luciferase and host mRNAs in different cells to those in parental U251 cells in the absence of IFN-γ, respectively. The experiments were repeated three times. The standard deviation is indicated by the error bar.

In the first set of experiments, we used a reporter system to study the effect of UL23 on the IFN-γ-induced transcription responses. The luciferase reporter plasmid pGL3-Promoter-3×GAS and the internal control reporter plasmid pRL-TK were transfected into different cells. At 24 hours post transfection, cells were treated with IFN-γ and cultured for additional 24 hours. As expected, treatment of IFN-γ resulted in the augment of IFN-γ-dependent transcription from the reporter construct in U251 and U251-C cells, which contained the control empty vector pCDNA (Fig 6A). However, much less increase of IFN-γ-dependent transcription was observed from the reporter construct in U251-UL23 cells that expressed UL23 (Fig 6A). In the second set of experiments, we examined the effect of UL23 on the expression of *HLA-B*, *IRF1*, and *IFIT3* genes, which are regulated by IFN-γ [10,24]. As shown in Fig 6B and 6D, the mRNA levels of these genes induced by IFN-γ were much less in U251-UL23 cells than those of parental U251 cells and control U251-C cells. These results suggest that UL23 inhibits the induction of the transcription of IFN-γ stimulated genes mediated by the interaction between Nmi and STAT1, possibly by modulating cellular localization of Nmi and STAT1.

**Fig 7:**
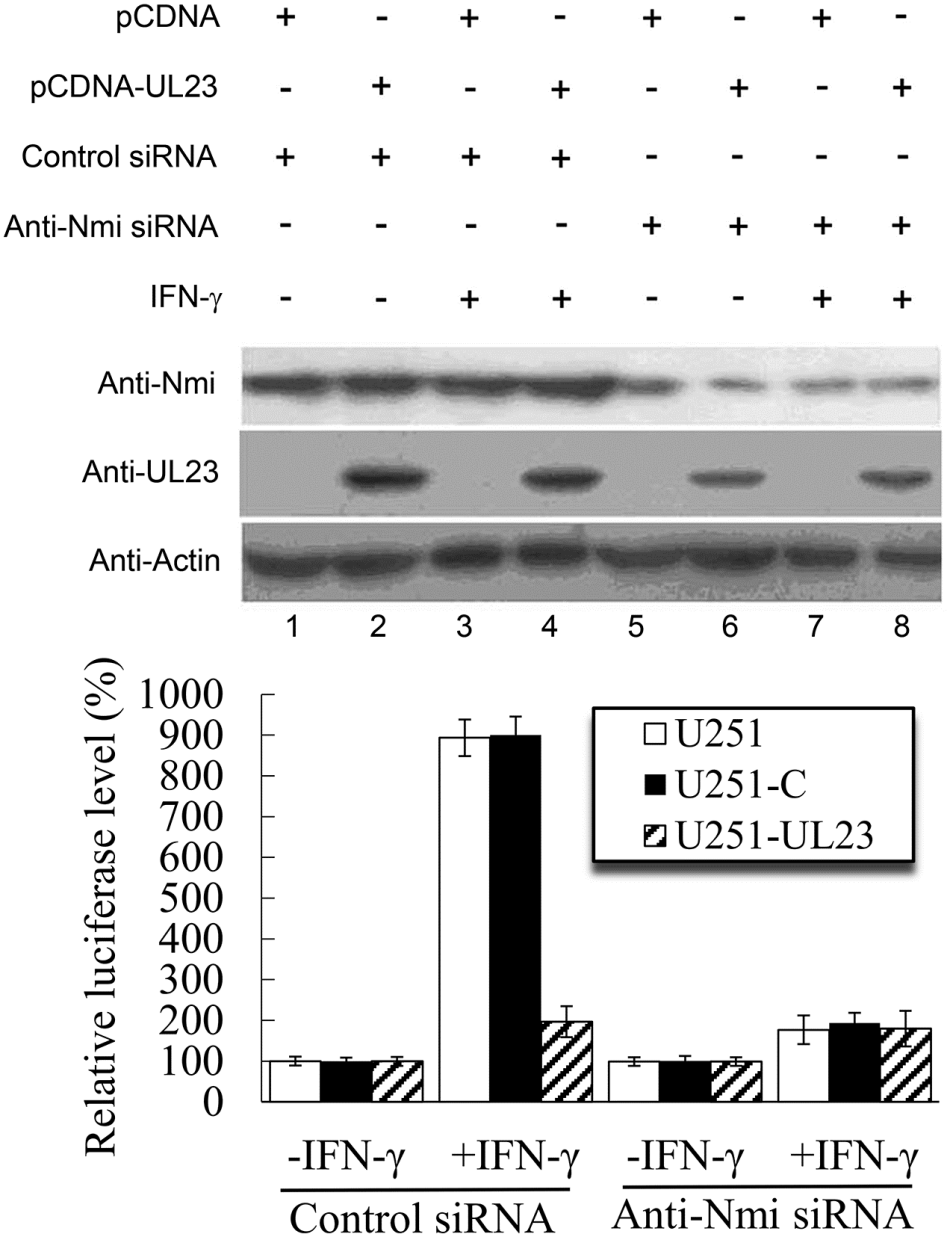
Role of Nmi in UL23-mediated inhibition of IFN-γ induced transcription. U251, U251-C, and U251-UL23 cells were treated with anti-Nmi siRNA and control siRNA, following the procedures described previously [59]. At 24 hours treatment, cells were transfected with reporter plasmid pGL3-Promoter-3×GAS and the internal control reporter pRL-TK. After 24 hours, cells were cultured in the absence (-IFN-γ) and presence (+IFN-γ) of IFN-γ (1000 U/ml) for 24 hours. The levels of UL23 and Nmi were determined by western blot analysis using those of actin as the loading control. Luciferase activity was determined luminometrically as relative light units (RLU). The values of the relative luciferase level represent the ratios of the levels of luciferase in different cells to those in parental U251 cells in the absence of IFN-γ. The experiments were repeated three times. The standard deviation is indicated by the error bar.

In the third set of experiments, we investigated the role of Nmi in the UL23-mediated inhibition of IFN-γ-induced transcription response. Different cells (e.g. U251, U251-C, and U251-UL23) were transfected with siRNA molecules that target the Nmi mRNA (anti-Nmi siRNA) or do not recognize any viral or cellular transcripts (control siRNA), respectively. Treatment with anti-Nmi siRNA substantially reduced the expression of Nmi in these cells (Fig 7, compare lanes 5–8 with lanes 1–4). At 24 hours after siRNA transfection, cells were further transfected with pGL3-Promoter-3×GAS and pRL-TK and treated with IFN-γ. Downregulation of Nmi expression mediated by siRNA decreased the IFN-γ-induced transcription in U251 and U251-C cells (Fig 7). However, anti-Nmi siRNA did not further decrease the IFN-γ-induced transcription in U251-UL23 cells, which expressed UL23 (Fig 7). These results are consistent with our results that UL23 modulates the level of IFN-γ-induced transcription by interacting with Nmi and disrupting its interaction with STAT1.

### Inhibition of IFN-γ induced transcription by UL23 in the context of HCMV infection

**Fig 8:**
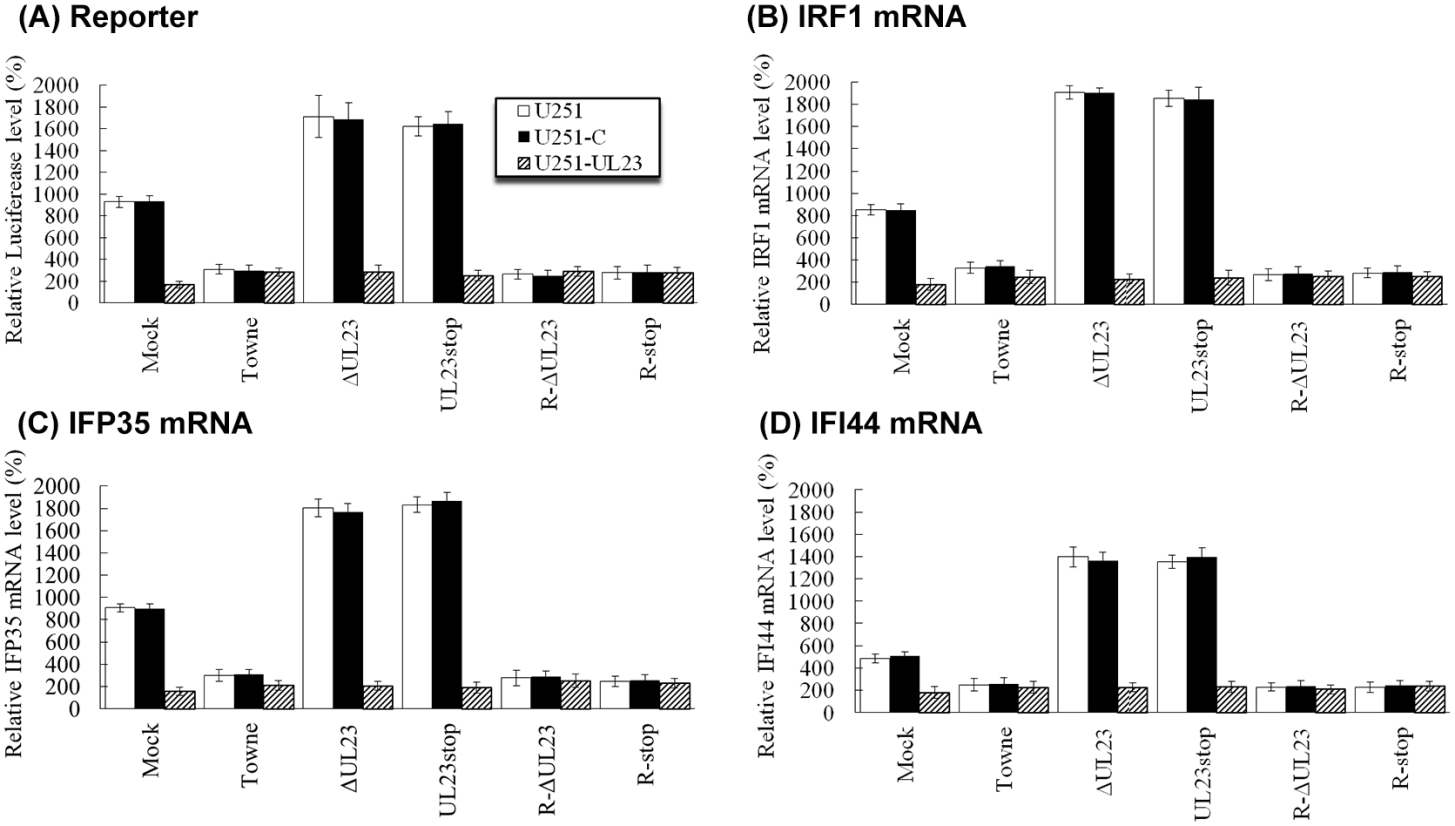
Inhibition of the transcription of IFN-stimulated genes by HCMV carrying functional UL23. (A) U251, U251-C, and U251-UL23 cells were transfected with reporter plasmid pGL3-Promoter-3×GAS and the internal control reporter pRL-TK. After 24 hours, cells were cultured in the absence (-IFN-γ) and presence (+IFN-γ) of IFN-γ (1000 U/ml). At 12 hours post-treatment, cells were mock-infected (mock) or infected with Towne_BAC_, UL23, UL23stop, R-UL23, or R-stop (MOI = 1) for 24 hours. Luciferase activity was determined luminometrically as relative light units (RLU). Only the results from experiments in the presence of IFN-γ are shown. (B-D) U251, U251-C, and U251-UL23 cells were cultured in the absence (-IFN-γ) and presence (+IFN-γ) of IFN-γ (1000 U/ml) for 12 hours, and then mock-infected or infected with different viruses. At 24 hours postinfection, total RNAs were extracted from cells. The levels of the *IRF1* (B), *IFP35* (C), and *IFI44* mRNAs (D) were determined by qRT-PCR using those of actin as the internal control. Only the results from experiments in the presence of IFN-γ are shown. The values of the relative luciferase and mRNA level represent the ratios of the levels of luciferase and host mRNAs in different cells treated with IFN-γ to those in parental U251 cells in the absence of IFN-γ, respectively. The experiments were repeated three times. The standard deviation is indicated by the error bar.

Our results suggest that UL23 inhibits IFN-γ induced transcription responses by interacting with Nmi. Hence, it is reasonable to suggest that viral mutants lacking UL23 expression may induce higher IFN-γ-dependent transcription and exhibit less resistance to IFN-γ than the parental virus and rescued viruses that express UL23. To determine if this is the case, cells were transfected with the luciferase reporter plasmid pGL3-Promoter-3×GAS and the internal control reporter plasmid pRL-TK, treated with IFN-γ, and then infected with different viral mutants. At 12 hours postinfection, treatment of IFN-γ led to significant induction of luciferase activity from the reporter construct in mock-infected cells as well as in cells infected with UL23-deficient mutants, ΔUL23 and UL23stop (Fig 8A). In contrast, infection of parental Towne_BAC_ and rescued viruses R-ΔUL23 and R-Stop, which expressed UL23 protein, suppressed the IFN-γ-mediated induction of the luciferase activity from the reporter construct (Fig 8A). The IFN-γ-mediated induction of the luciferase activity as observed in mock-infected U251 cells and cells infected ΔUL23 and UL23stop was not found in mock-infected U251-UL23 cells and U251-UL23 cells infected with these viral mutants, suggesting that UL23 expression inhibited the IFN-γ-mediated induction (Fig 8A). Similar results were also observed when the transcription levels of IFN-γ stimulated genes *IRF1*, *IFP35*, and *IFI44* were assayed in cells infected with these viruses (Fig 8B and 8D). These results suggest that IFN-γ induced transcription can be inhibited by UL23 in the context of HCMV infection.

### Inhibition of antiviral IFN-γ response and increased viral resistance to IFN-γ by UL23 in the context of HCMV infection

**Fig 9:**
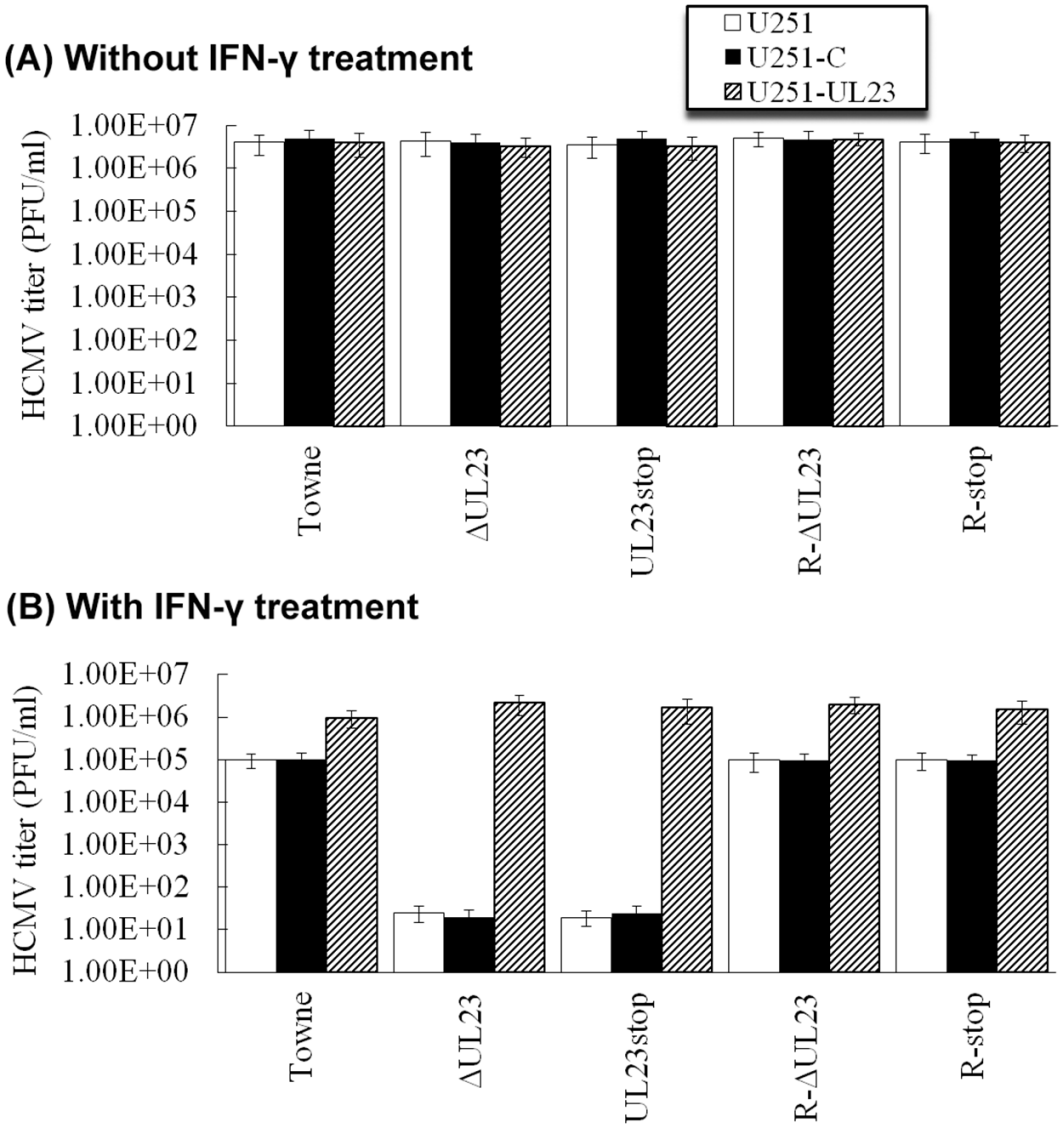
HCMV growth in different cells that were treated with and without IFN-γ. U251, U251-C, and U251-UL23 cells were incubated in the absence (-IFN-γ)(A) and presence (+IFN-γ)(B) of IFN-γ (1000 U/ml) for 12 hours, and then mock-infected or infected with Towne_BAC_, ΔUL23, UL23stop, R-UL23, and R-stop (MOI = 1). Total infection cultures were collected at 5 days postinfection and viral titers were determined [39]. The analyses were repeated three times and the standard deviation is indicated by the error bar.

In our experiments, a reduction of at least 50 fold in viral titers was found in IFN-γ-treated Towne-infected cells compared to those untreated cells (compare Towne-infected U251 cells in Fig 9A and 9B), consistent with previous observations that HCMV replication and infection is inhibited in IFN-γ-treated cells [4,10,50]. If UL23 blocks IFN-γ induced transcription responses during HCMV infection, it is conceivable that HCMV mutants lacking UL23 expression may be more susceptible to IFN-γ treatment and exhibit less growth than the parental and rescued viruses that express UL23. To determine if this is the case, different cells were either pretreated with IFN-γ or not, and then infected with viruses. Growth of different viral mutants in these cells was studied. In the absence of IFN-γ treatment, mutants ΔUL23 and UL23stop exhibited similar titers as the parental strain Towne_BAC_ and the control revertant viruses R-ΔUL23 and R-stop (Fig 9A), consistent with previous observations that UL23 is dispensable for viral lytic infection [39]. However, upon treatment of IFN-γ, UL23-minus mutants, ΔUL23 and UL23stop, exhibited a peak titer of at least 5,000 fold lower than Towne_BAC_ and revertant viruses R-ΔUL23 and R-stop in U251 and U251-C cells (Fig 9B). To further determine the role of UL23, we repeated the experiments using U251-UL23 cells. We observed no difference in the titers of Towne_BAC_, ΔUL23, UL23-stop, R-ΔUL23, and R-UL23-stop in U251-UL23 cells pre-treated with IFN-γ (Fig 9B). These results suggest that UL23 blocks IFN-γ induced response and increases viral resistance to IFN-γ during viral infection, possibly by interacting with Nmi and modulating the cellular localization of STAT1 protein.

## Discussion

IFN represents one of the most important innate immunity responses in a host to combat infections of many human viruses [11,24]. Understanding the mechanism of how human viruses modulate IFN responses to achieve successful infection will facilitate the development of novel strategies for the treatment and prevention of human viral infections. In this study, we provide the first direct evidence that HCMV UL23 protein specifically interacts with human N-myc interactor (Nmi) protein. We identified this interaction through a yeast two-hybrid screen and co-immunoprecipitation in human cells. We also showed that Nmi, when bound to UL23, was not associated with STAT1, suggesting that UL23 binding of Nmi disrupts the interactions of Nmi with STAT1. In IFN-γ treated cells overexpressing UL23, we observed (a) significant reduced levels of Nmi and STAT1 in the nuclei, the sites where these proteins act to induce transcription of IFN-γ stimulated genes, and (b) decreased levels of the induction of the transcription of IFN-γ stimulated genes. UL23-deficient HCMV mutants induced higher transcription of IFN-γ stimulated genes and exhibited lower titers than parental and control revertant viruses expressing a functional UL23 in IFN-γ treated cells. These results suggest that UL23 functions to bind to Nmi and reduce the nuclear import and the availability of Nmi and its associated protein STAT1 for the activation of IFN-γ stimulated gene transcription, leading to a decrease of IFN-γ dependent responses and an increase of viral resistance to IFN-γ.

It is possible that Nmi may bind non-specifically to tagged or over expressed proteins but not the native viral protein. However, several experimental results presented in our study indicate that this is not the case and that the observations from using the tagged UL23 protein may be identical to those using the native untagged protein. First, in co-immunoprecipitation experiments, Nmi interacted with the HA-tagged UL23 but not the negative control, UL70, suggesting that Nmi does not bind to the tag sequence (Fig 1A and 1B). Second, Nmi co-immunoprecipitated with untagged native UL23 but not UL44 in HCMV-infected cells, demonstrating that the interaction with UL23 during HCMV infection is specific (Fig 1C and 1D). Third, both tagged and untagged UL23 protein co-localized with Nmi (Figs 4 and 5, S2 Table). These results suggest that (1) the tag sequence does not enhance or interfere with the interaction of Nmi and UL23, (2) the interaction is specific, and (3) the observations from experiments with the tagged UL23 protein may be representative of those obtained with the native untagged protein during HCMV infection.

IFN-γ response is one of the most important components in the immune responses against human viruses including HCMV [11,24]. IFN-γ induces transcription of many IFN-stimulated genes such as those involved in antigen presentation and immunomodulation, conferring increased antiviral effects of the stimulated cells [10]. Nonprofessional antigen presenting cells have been shown to express MHC II molecules upon IFN-γ stimulation and subsequently present viral antigens including HCMV IE1 peptides [51]. IFN-γ produced by CD4+ T cells can block HCMV replication in vitro, highlighting the important role of IFN-γ in controlling HCMV infection [50]. It has been further shown that IFN-γ is more potent than IFN-α or -β against HCMV and murine cytomegalovirus (MCMV), and type I and II IFNs act synergistically when administered in combination [10]. Consistent with these previous observations, our experiments showed that a reduction of more than 50 fold in viral titers was observed in HCMV infected cells treated with IFN-γ compared to those untreated cells (Fig 9).

In order to escape from and counteract against IFN responses, many human viruses including herpesviruses such as HCMV and MCMV have been shown to modulate various steps of IFN induced signaling pathways by encoding immunomodulatory proteins [11,24]. By targeting different steps during the IFN response at different time points of the infection, viruses can counteract IFN responses by interfering with the IFN downstream signaling and become resistant against existing IFN. Viruses can also express evasins targeting IFN induction within the infected cells to avoid the priming of bystander cells. For example, HCMV IE1 proteins have been shown to bind with STAT proteins (e.g. STAT2) and these interactions diminish DNA binding of the ternary ISGF3 complex to promoters of type I ISGs, leading to inhibition of transcription activation of human IFN stimulated genes including *ISG54*, *PKR*, and *CXCL10* [31,34–37]. Recent studies have also shown that IE1 can elicit a Type II interferon-like host cell response that depends on activated STAT1 but not IFN-γ [35]. Moreover, it has been recently reported that IE1 can rewire upstream IL6-type to downstream IFN-γ-like signaling, two pathways linked to opposing effects, resulting in repressed STAT3- and activated STAT1-responsive genes [52]. MCMV also encodes a viral protein, M27, which modulates IFN signaling and confers IFN resistance [53]. M27 can bind to STAT2 protein and block STAT2-mediated IFN-γ signaling and antiviral responses by recruiting DNA-damage DNA-binding protein (DDB) 1, a host ubiquitin ligase complex adaptor protein, for targeting STAT2 for proteasome degradation [53,54]. However, no proteins encoded by herpesviruses including CMV have been reported to interact with Nmi and modulate Nmi-mediated immune responses. Our study provides the first direct evidence that a herpesvirus encodes a viral protein that specifically binds to Nmi and modulates signaling steps of IFN-γ responses mediated by Nmi.

In response to interferon and cytokine signaling, Nmi is a transcription cofactor that can modulate the activity of members of the STAT protein family [55] and has been shown to play a central role in IFN-γ induced STAT1-dependent transcription [30]. During Sendai virus infection, Nmi can bind to IRF7, a master regulator for Type I IFN-dependent immune responses, and target IRF7 for proteasome-mediated degradation, leading to inhibition of Type I IFN responses induced by viral infections [56]. In recent studies, a miRNA encoded by torque teno virus (TTV) targets Nmi expression to inhibit type II interferon signaling and promote immune evasion [57] while protein 6 of severe acute respiratory syndrome coronavirus (SARS-CoV) can bind to Nmi and promote its degradation [58]. These results suggest the critical roles of Nmi in controlling infections of these viruses and possibly, other human viruses. Consistent with these observations, we showed (a) that over-expression of UL23 modulated the cellular localization of Nmi and its associated protein STAT1 and inhibited IFN-γ signaling mediated by Nmi and STAT1 and (b) that UL23-deficient mutants induced much higher IFN-γ responses and were more susceptible to IFN-γ antiviral effects. These results highlight the important roles of Nmi in human anti-HCMV immune responses and roles of UL23-Nmi interactions in conferring resistance to IFN-γ responses.

Little is currently known about the effects UL23 may have on the resistance of HCMV to type I compared to type II IFNs. IFN-γ has been shown to be more potent than IFN-α or -β against HCMV and MCMV, and type I and II IFNs act synergistically when administered in combination [10,31,34–37]. As Nmi interacts with multiple STAT proteins, it is conceivable that UL23 may affect the expression of IFN-α/β responsive genes in addition to the expression of IFN-γ responsive genes as shown in this study (Figs 6 and 8). Our preliminary studies suggest that UL23 modulates the expression of several IFN-α/β responsive genes. Further studies on these issues will elucidate the roles of UL23 in interfering with host type I and type II IFN responses and facilitating HCMV infection and replication.

It is possible that Nmi interacts with HCMV proteins other than UL23, leading to a modulation of IFN-γ stimulated gene transcription and IFN-mediated responses as observed in our experiments. However, we observed no positive interaction between Nmi and more than 100 HCMV ORFs other than UL23 in our yeast two hybrid screen experiments [44]. UL23-mediated inhibition of IFN-γ induced gene transcription can be mimicked by suppressing Nmi expression using anti-Nmi siRNAs (Fig 7), and UL23-deficient viral mutants (i.e. ΔUL23 and UL23stop) exhibited significantly more IFN-γ responses than parental virus Towne_BAC_. While we cannot completely exclude the possibility that Nmi may affect the IFN-γ responses by interacting with HCMV proteins other than UL23, our results in experiments expressing UL23 alone in the absence of HCMV infection and in experiments using anti-Nmi siRNA and UL23-deficient mutants suggest that UL23 may interact with Nmi and affect its cellular localization and interactions with STAT1 in the absence of other HCMV proteins, possibly leading to the inhibition of IFN-γ induced STAT1-dependent transcription. The coding sequences of more than 30 human proteins other than Nmi were also found to interact potentially with UL23 in our yeast two hybrid assays (S1 Table). To confirm the interactions of these proteins with UL23, we will perform additional experiments (e.g. co-immunoprecipitation) and these results will elucidate the effects of other human proteins on UL23 function during HCMV infection.

We showed that UL23 interacted with Nmi in co-IP experiments in various cell types, including U251, human foreskin fibroblasts, and HeLa cells. Furthermore, U251 cells were used to investigate the roles of UL23 in Nmi localization and HCMV infection. It may be important to investigate the function of UL23 in fibroblasts, which are fully permissive and commonly used to study HCMV infection [4]. However, due to poor transfection efficiency in fibroblasts, it is technically difficult to carry out similar experiments in these cells. Moreover, it is not feasible to constitutively express high level of UL23 over many passages in fibroblasts because they are primary cells. In contrast, the neuronal origin U251 cells are permissive to HCMV infection and have been used to study HCMV previously [4,59,60]. As these cells are immortalized, U251 cell lines that constitutively express UL23 can be easily generated. Additional investigation in other cells such as fibroblasts will facilitate the elucidation of UL23 function.

Our results indicate that overexpression of UL23 impedes the nuclear translocation of Nmi and retains it in the cytoplasm. The mechanism of cytoplasmic retention of Nmi by UL23 is currently unknown. Nmi has been shown to interact with several transcription factors such as the Myc family members and STATs, and is primarily localized in the nuclei under certain conditions (e.g. under cellular stress and IFN treatment) [25,30,49]. These observations are consistent with our results that Nmi is predominantly localized in the nuclei of IFN-γ treated cells (Fig 4). Meanwhile, Nmi has been reported to localize in the cytoplasm in untreated cells and the domains of Nmi that facilitate nuclear import and cytoplasmic localization have been studied [45]. However, little is known about how Nmi is imported into the nuclei and the protein carriers for its nuclear transport have not been reported.

It is conceivable that UL23 sequesters Nmi in specific cytoplasmic compartments so that Nmi is not available for interaction with other viral or cellular protein carriers for nuclear transport. It is currently not known how binding of Nmi by UL23 inhibits the association of Nmi with STAT1. Previous studies have shown that the STAT binding domains of Nmi are in amino acids 57–99 and 143–202 [30]. The minimal Nmi mutant that binds to UL23 contains amino acid 199 to 292 and covers the NID2 domain critical for Nmi homo-and hetero-dimerization [40,47], which partially overlaps with the binding domains to STAT proteins (Fig 2). Perhaps UL23 may compete for binding to the same Nmi regions as STAT1. It is also possible that binding of UL23 may affect the conformation of Nmi, leading to inhibition of its binding to STAT1.

HCMV IE1 proteins have been shown to bind with STAT proteins (e.g. STAT2) and these interactions diminish DNA binding of the ternary ISGF3 complex to promoters of type I ISGs, leading to inhibition of transcription activation of human IFN stimulated genes including *PKR*, *CXCL10*, and *ISG54* [31,34–37]. Recent studies have also shown that IE1 can elicit a Type II interferon-like host cell response that depends on activated STAT1 but not IFN-γ [35]. Moreover, it has been recently reported that IE1 can rewire upstream IL6-type to downstream IFN-γ-like signaling, two pathways linked to opposing effects, resulting in repressed STAT3- and activated STAT1-responsive genes [52]. The common impacts on STAT1 by IE1 and UL23 via different pathways, represent an interesting and potentially important regulatory mechanism of host responses during HCMV infection. UL23 encodes a tegument protein and IE1 is an immediately early protein [31,34–38]. HCMV IE1 is dispensable for replication of a UL23 competent virus under high multiplicity of infection (MOI) conditions while UL23 is not essential for replication of an IE1 competent virus in cultured cells [39, 61]. It is possible that UL23-mediated modulation of STAT1 signaling is enhanced or repressed by IE1-mediated effect on STAT1 function. Further studies on these issues may elucidate the potential roles of the impact on the STAT1 signaling by these two HCMV proteins in supporting HCMV infection.

How STAT1 protein is retained in the cytoplasm in the presence of UL23 is currently unknown, given the fact that UL23 binds to Nmi and disrupts Nmi interaction with STAT1. While phosphorylation of STAT1 is required for its nuclear localization, the mechanism of the nuclear import of STAT1 is not completely understood [20,23,24]. Our results showed that Nmi and STAT1 interacted with each other and localized in the nuclei in IFN-γ treated cells. However, in the presence of UL23, these two proteins failed to interact with each other and were localized in the cytoplasm (Figs 4 and 5). It is conceivable that Nmi or Nmi-associated proteins facilitate STAT1 nuclear import by recruiting STAT1 to the Nmi-containing complex that may contain the protein carriers for STAT1 nuclear import. Binding of Nmi by UL23 disrupts the interactions of STAT1 with Nmi, and may block the association of STAT1 with the Nmi-containing complex that contains the protein carriers for STAT1 nuclear import, leading to its accumulation in the cytoplasm. Detailed characterization of the nuclear import processes of Nmi and STAT1 proteins should clarify these issues. Further studies on the interactions of UL23 with Nmi and the effects of these interactions on Nmi-associated proteins will provide insight into the mechanism of how Nmi and its mediated immune responses play a role in combatting viral infections in a host and how viruses such as HCMV develop novel strategies to escape from or counteract against these immune responses including those Nmi-mediated IFN-γ responses.

## Materials and methods

### Ethics statement

This study was carried out in strict accordance with the recommendations in the Guide for the Care and Use of Laboratory Animals of the National Institutes of Health (8^th^ Edition). The protocol for all animal experiments was either approved by the Animal Care and Use Committees of the University of California-Berkeley (Protocol #R240) or Jinan University (Guangzhou, China). All efforts were made to minimize suffering.

### Cell, viruses, and antibodies

Human glioblastoma U251 cells, HEK 293T cells, and Hela cells were purchased from American Type Culture Collection (ATCC) (Manassas, VA) while human foreskin fibroblasts (HFFs) were obtained from Lonza Inc (Allendale, NJ) [62]. HCMV (Towne_BAC_, ΔUL23, UL23stop, R-ΔUL23, and R-stop) was grown in human cells [39].

Rabbit anti-FLAG (Santa Cruz Biotech, Santa Cruz, CA), mouse anti-β-actin (Proteintech, Manchester, United Kingdom), mouse anti-HA (Proteintech), mouse anti-Nmi (Santa Cruz Biotech), and mouse anti-STAT1 (BD Biosciences, San Jose, CA) antibodies were used in the experiments. Mouse monoclonal anti-FLAG antibody was purchased from Cell Signaling Technology, Inc (Boston, MA). Cy3- or FITC-labeled secondary antibodies were purchased from Beyotime (Shanghai, China) and Invitrogen (Carlsbad, CA), respectively. The anti-UL44 (Virusyn, Taneytown, MD) and anti-IE1 HCMV monoclonal antibodies used in this study have been described previously [60]. UL44 was detected as a predominant species in HCMV-infected cells using the antibody [60,63].

The anti-UL23 monoclonal antibody was generated by cloning the UL23 sequence into plasmid pET-28a(+) (Novogen, Madison, WI). His-tagged UL23 protein isolated from *E*. *coli* BL21 (DE3) was used to immunize BALB/C mouse. The spleen cells were subsequently isolated and fused with myeloma cells. ELISA screening and HAT culture medium were used to select for positive fused cells. Positive hybridoma cell cultures were propagated to generate UL23 monoclonal antibody. Western blot analyses, co-IP, and indirect immunofluorescence assays were subsequently used to characterize and confirm the reactivity of the antibody to UL23 protein. Special efforts were made to screen many clones to identify those clones that generated anti-UL23 antibodies with excellent reactivity and specificity. For example, to assess their specificity, antibodies were serially diluted and screened for their reactivity with lysates of uninfected cells and cells infected with HCMV Towne and ΔUL23. Only those anti-UL23 antibodies that reacted with lysates of cells infected with Towne but not with those of uninfected cells or cells infected with ΔUL23 were used in our study.

To generate U251-FLAG, U251-FLAG-UL23, U251-C, and U251-UL23 cell lines, DNAs of constructs pCMV-FLAG, pCMV-FLAG-UL23, pCDNA, and pCDNA-UL23 were co-transfected into U251 cells with DNA of LXSN vector [64]. Neomycin with a final concentration of 600 μg/ml was added to the culture medium at 48–72 hour postinfection and neomycin resistant cells were selected and cloned after two weeks [65,66]. The levels of UL23 in individual cell clones were determined by Western blot analysis.

### Plasmid constructs

The constructs generated in this study are listed in Table 1. The construct containing the DNA sequence of Nmi was purchased from Origene Technologies, Inc (Montgomery, MD). Constructs pGBKT7-UL23 and pCMV-HA-UL23 that were used for the yeast two hybrid screen and for the expression in human cells have been previously described [44]. To generate pGL3-Promoter-3×GAS, the coding sequence of GAS was constructed by using oligonucleotide GASsense (5’-CAATTCTGTGAAGAAAGAATTCTGTGAAGAAAGAATTCTGTGAAGAAAG-3’) and oligonucleotide GASantisense (5’-GATCTCTTTCTTCACAGAATTCTTTCTTCACAGAATTCTTTCTTCACAGAATTGGTAC- 3’). To generate pGADT7-Nmi, the coding sequence of Nmi was amplified by PCR using the primers Nmi-F1 (5’-AAG- CATATGGAAGCTGATAAAGATGAC-3’) and Nmi-R (5’-GAACTCGAGTT- CTTCAAAGTATGCTATGTG-3’) from a human embryo kidney cDNA library of Matchmaker two-hybrid system 3 (Clontech, South San Francisco, CA) and then cloned into 5’NdeI/3’Xho I-digested pGADT7. To generate pCMV-FLAG-Nmi, the coding sequence of Nmi was amplified by PCR using the primers Nmi-F1 (5’-AAGGAATTCGAAGCTGATAAAGATGAC-3’) and Nmi-R (5’-GAACTCGAGTTCTTCAAAGTATGCTATGTG-3’) and then cloned into 5’EcoRI/3’XhoI-digested pCMV-FLAG. Various designed UL23 deletion mutant DNA sequences were generated by PCR using pGBKT7-UL23 as the template. The PCR products were cloned into NdeI/BamHI-digested pGBKT7 for yeast two hybrid analysis and SalI/KpnI-digested pCMV-FLAG for expression in human cells. The constructs for various Nmi deletion mutants were generated by PCR using pGADT7-Nmi as the template. The PCR products were cloned into NdeI/BamHI-digested pGADT7 for yeast two hybrid analysis and SalI/KpnI-digested pCMV-FLAG for expression in human cells. All constructs were subjected to restriction digestion profile and sequencing analysis for confirmation.

### Yeast two-hybrid

A cDNA library derived from human embryonal kidney (Clontech) was screened using the Matchmaker two-hybrid system 3 (Clontech, South San Francisco), following the procedures as described previously [44,62].

### Western blotting and co-immunoprecipitation assay

Cell lysates were obtained after incubating cells with RIPA lysis buffer (Sigma Aldrich, St. Louis, MO) supplemented with protease inhibitor cocktail (Roche, Basel, Switzerland). The protein content was determined by Bradford assay (Bio-Rad, Hercules, CA). Equivalent amounts of protein were separated by SDS-PAGE, transferred onto membranes, reacted with antibodies, stained using a Western chemiluminescent substrate kit (Thermo Fisher, Waltham, MA) and quantitated with a STORM840 PhosphorImager or a Gel Documentation Station (BioRad, Hercules, CA) [60,64]. The samples were serially diluted and analyzed in order to accurately determine the protein levels in the cytoplasm or nuclei. The percentages of STAT1 and Nmi were calculated using the levels of actin or histone H1 in cytoplasm and in nuclei as the internal controls, respectively. The experiments were repeated three times.

Co-IP experiments were performed using the protein A/G immunoprecipitation kits (Cell Signaling Technology, Inc, Boston, MA) [44]. To perform Western blot staining of immunoprecipitated protein samples (e.g. in Figs 1, 3, and 5), the primary antibodies were directly conjugated to alkaline phosphatase (AP) with an AP conjugation kit (Abcam, Cambridge, MA) and then purified, following the manufacturer’s recommendation. The immunoprecipitated protein samples were separated by SDS-PAGE and transferred onto membranes, reacted with the AP-conjugated primary antibodies in the absence of secondary antibodies, and stained using a Western chemiluminescent substrate kit (Thermo Fisher, Waltham, MA) and quantitated with a STORM840 PhosphorImager or a Gel Documentation Station (BioRad, Hercules, CA) [60,64].

### Luciferase analyses assay

A luciferase reporter assay (Promega, Madison, WI) was used to evaluate the activation of the IFN-γ signal pathway. Luciferase activity was quantified using a luminometer (TD-20/20; Turner Designs, Sunnyvale, CA) according to the manufacturer’s instructions. Briefly, cells were seeded into 6-well plates and transiently transfected or electroporated with reporter plasmid pGL3-Promoter-3×GAS, Renilla luciferase expression plasmid pRL-TK (Promega, Madison, WI), and the indicated expression plasmids using the PolyFect Transfection system (Qiagen). At 24 hours posttransfection, cells were mock-treated or treated with IFN-γ (1000 U/ml) (R&D, Minneapolis, MN) for 24 hours, lysed in passive lysis buffer of Dual-Luciferase Reporter Assay System (Promega, Madison, WI), and the luciferase readings of each sample were normalized against the rLuc levels. Experiments were repeated three times. All of the data shown in this study were obtained from at three independent experiments.

### Transfection of siRNA into cells

Cells were seeded in 6 well plates and transfected with the corresponding siRNA using Lipofectamine 2000 (Invitrogen, Carlsbad, CA), according to the manufacturer’s instructions. Small interfering RNAs (siRNAs) targeting human Nmi mRNA and control siRNAs were designed and synthesized by Thermo Fisher Scientific (Waltham, MA). Cells were transfected with siRNAs as described previously [59], and then mock-treated or treated with IFN-γ (1000 U/ml) (R&D, Minneapolis, MN) for the indicated time. The cell lysates were further assayed for luciferase activities or western blotting.

### Immunofluorescence assays

Cells grown on glass coverslips, which were treated with IFN-γ and mock-infected or infected with different viruses for different periods of time, were washed with washing buffer (0.1% BSA in phosphate-buffered saline (PBS)), fixed and permeabilized with PBS containing 4% paraformaldehyde and 0.2% Triton X-100. Cells were then incubated with appropriate primary antibodies and secondary antibody Cy3 IgG or FITC IgG (Beyotime, Shanghai, China). Nuclear staining was performed with 4’,6-diamidino-2-phenylindole (DAPI) (Beyotime, Shanghai, China). The cells were visualized with a Nikon Eclipse TE2000-S microscope. Confocal images were collected using a ZEISS LSM700 microscope in separate channels and the digital images were subsequently merged by using the ZEN software (ZEISS, Oberkochen, Germany). In some of the experiments, images were taken with a Nikon Eclipse TE300 microscope using a SPOT RT Slider camera and imaging software (Diagnostic Instruments, Inc., Sterling Heights, MI) [62]. We manually counted cells showing UL23, Nmi, and STAT1 localization and carried out three independent experiments to assay the percentages of the numbers of cells.

### Cytoplasmic and nuclear extract preparation

Cells were harvested and suspended in buffer A (10 mM HEPES (pH 7.4), 10 mM KCl, 1 mM dithiothreitol, 0.6% NP-40) (TransGen Biotech, Shanghai, China) [59]. We collected the supernatant as the cytoplasmic fraction after centrifugation. We suspended the remaining pellet in nuclear extraction buffer (20 mM HEPES (pH 7.4), 150 mM NaCl, 1 mM dithiothreitol) (TransGen Biotech, Manchester, United Kingdom), and collected the supernatant as the nuclear fraction after centrifugation [59]. We used equal amounts of cytoplasmic and nuclear extracts for immunoblotting of STAT1, Nmi, actin, and histone H1 (Proteintech, Manchester, United Kingdom).

### RNA isolation and quantitative reverse transcription-PCR (qRT-PCR)

Quantitative reverse transcription-PCR (qPCR) analysis of viral mRNA was carried out as described elsewhere [67]. Total RNA was extracted from cells with TRIzol (Invitrogen, Carlsbad, CA) according to the manufacturer’s manual. RNA (500 ng) was reverse transcribed using a cDNA synthesis kit (TAKARA, Shanghai, China) and PCR reactions were performed using SYBR Green supermix (Applied Biosystems, Foster City, CA). Oligonucleotide primers used are as follows: *HLA-B* forward (5′-CTACCCTGCGGAGATCAC-3′), *HLA-B* reverse (5′-TAGGACAGCCAGGCCAGCAACA-3′); *IRF1* forward (5’-CGATACAAAGCAGGGGAAAA-3’), *IRF1* reverse (5’-GTGGAAGCATCCGGTACACT-3’); *IFIT3* forward (5’-TTCTCCTCTGGACTGGCAAT-3’), IFIT3 reverse (5’-AGGACATCTGTTTGGCAAGG-3’); *IFP35* forward (5’-CTAGGGATGGAGTGGCTCAG-3’), *IFP35* reverse (5’-TCAGGAATGTTGAGCACCAG-3’); *IFI44* forward (5’-AGCCGTAGTGGGGTCTGATA-3’), *IFI44* reverse (5’-ATGTGGGGAATGTCATCCAT-3’); β-actin forward (5’-TCGTCCACCGCAAATGCTTCTAG-3’), β-actin reverse (5’-ACTGCTGTCACCTTCACCGTTCC-3’). Thermal cycling conditions were as follows: 50°C for 2 min, 95°C for 15 min, and 45 cycles of 95°C for 30 sec and 60°C for 1 min [59]. Relative quantitation was determined using the comparative CT method with data normalized to β-actin. The PCR results were derived from three independent experiments.

### Construction of viral mutants, viral infection, and virus growth analysis

Mutant ΔUL23, which contained a deletion of the entire coding sequence of UL23, was derived from Towne_BAC_ and has been previously described [39]. A two-step mutagenesis protocol was used to construct mutant UL23stop, which contained a stop codon immediately downstream from the UL23 translation initiation site. In the first step, we inserted at the UL23 translation initiation codon with a cassette (tet/str) containing the tetracycline resistance gene tetA and rpsL gene conferring streptomycin susceptibility, following the mutagenesis procedures as described previously [39]. The bacteria harboring the mutant BAC constructs were electroporated with the PCR-amplified tet/str cassette. Successful insertion of the tet/str cassette was screened by selecting for bacterial colonies resistant to tetracycline. In the second step, the tet/str cassette was targeted for deletion and replacement by the mutated UL23 sequence that contained a stop codon immediately downstream from the translation initiation site. The resulting mutant, which only contained the mutated UL23 sequence and would not contain the cassette, was streptomycin-resistant and, therefore, was easily selected in the presence of the antibiotics [39]. Rescued mutants R-ΔUL23 and R-stop were generated from ΔUL23 and UL23stop, respectively, following the experimental procedures for construction of rescued viruses as described previously [39]. The UL23 region in the mutants and the rescued viruses was analyzed by restriction digestion profile and sequencing analyses. The expression of UL23 in cells infected with these mutants was studied by Western blot analysis using the anti-UL23 antibody.

Cells (n = 1x10^6^) were either mock-infected or infected with HCMV (MOI = 1–3) as described previously [39]. For quantification of viral growth, the cells and medium were harvested at 5 days postinfection and viral stocks were prepared. Viral stocks were serial diluted and used to infect 1 x 10^5^ human foreskin fibroblasts, followed by agar overlay. Viral titer was determined by counting the number of plaques 10–14 days after infection [39]. Susceptibility to IFN-γ was assayed by virus growth in the presence of human recombinant 1000 U/mL IFN-γ (R&D, Minneapolis, MN) after preincubation with IFN-γ for 12 hours before infection. The values obtained were averages from three independent experiments.

## Supporting information

S1 Fig Identification of the interactions between UL23 and Nmi and their derived deletion mutants by yeast two-hybrid screen. We co-transformed yeast strain AH109 with one BD and one AD plasmid as indicated. We first grew the transformed yeast cells containing both plasmids on SD-minus Trp/Leu plates (DDO) to maintain the two plasmids, then plated the colonies onto SD-minus Trp/Leu/Ade/His plates (QDO) and also subjected them to beta-gal activity test (x-gal) by filter lift staining. Experimental details are described in Methods and Materials. (A) No interactions were identified between AD-lam and AD-T antigen, which served as a negative control. Positive interactions were observed between BD-p53 and AD-T, which served as a positive control. (B-G) Interactions among the empty vectors AD-vector and BD-vector and the AD and BD constructs that contained the sequences of Nmi, UL23, and their deletion mutants.
(TIF)

S1 Table Gene sequences contained in the positive clones identified in the yeast two hybrid screens using the HCMV UL23 sequence. (PDF)

S2 Table The percentages of the numbers of cells in which Nmi and STAT1 were found to be localized in the nuclei (nuclei), cytoplasm (cytoplasm), or both (nuclei/cytoplasm). Different cells were treated with IFN-γ (1000 U/ml) and then either mock-infected or infected with Towne_BAC_, ΔUL23, R-ΔUL23, UL23stop, or R-stop at 12 hours post-treatment. At 24 hours post-infection, we stained cells with DAPI, anti-UL23, anti-Nmi, or anti-STAT1, and visualized the cells under a microscope. The experimental procedures were described in Materials and Methods.
(PDF)
