# Supplementary material for: Human cytomegalovirus UL23 inhibits transcription of interferon-γ stimulated genes and blocks antiviral interferon-γ responses by interacting with human N-myc interactor protein
Source: PLoS Pathog. 2018 Jan 29;14(1):e1006867. doi: 10.1371/journal.ppat.1006867 (PMC5805366; doi:10.1371/journal.ppat.1006867)
Supplement: S1 Table — (PDF) [file ppat.1006867.s002.pdf]

SUPPORTING INFORMATION

S1 Table. Gene sequences contained in the positive clones identified in the yeast two hybrid screens using the HCMV UL23 sequence.

Number	Gene name	ACCESSION Number
1	histidine triad nucleotide binding protein 1 (HINT1)	NM_005340
2	ribosomal protein L8 (RPL8)	NM_000973
3	keratin 18 (KRT18)	NM_000224
4	gelsolin (amyloidosis, Finnish type) (GSN)	NM_198252
5	MORC family CW-type zinc finger 4 （MORC4)	NM_024657
6	HIV-1 Rev binding protein	NM_004504
7	LUC7-like	NM_018032
8	insulin-like growth factor binding protein 4	NM_001552
9	ATPase inhibitory factor 1, nuclear gene encoding mitochondrial protein	NM_016311
10	chaperonin containing TCP1, subunit 7 (eta) (CCT7)	NM_001009570
11	testis derived transcript (3 LIM domains) (TES),	NM_015641
12	radical S-adenosyl methionine domain containing 1 (RSAD1)	NM_018346
13	interferon-induced protein 35 (IFI35)	NM_005533
14	chloride intracellular channel 1 (CLIC1)	NM_001288
15	aminoacylase 1 (ACY1)	NM_000666
16	leucine-rich repeats and calponin homology (CH) domain containing 3 (LRCH3),	NM_032773
17	DnaJ (Hsp40) homolog, subfamily B, member 6 (DNAJB6), transcript variant 2	NM_005494
18	eukaryotic translation initiation factor 3, subunit E(EIF3E)	NM_001568
19	metallothionein 1A (functional) (MT1A)	NM_005946
20	proteasome (prosome, macropain) subunit, beta type, 4 (PSMB4),	NM_002796
21	WW domain containing adaptor with coiled-coil (WAC), transcript variant 3	NM_100486
22	collagen, type I, alpha 2 (COL1A2),	NM_000089
23	N-myc and STAT interactor	NM_004688
24	mitochondrial ribosomal protein S9 (MRPS9)	NM_182640
25	zinc finger protein 219 (ZNF219)	NM_016423
26	enoyl Coenzyme A hydratase domain containing 1	NM_018479
27	versican (VCAN)	NM_004385
28	solute carrier family 16 (monocarboxylic acid transporters), member 3 (SLC16A3)	NM_004207

29	insulin-like growth factor binding protein 2	NM_000597
30	toll-like receptor adaptor molecule 1 (TICAM1)	NM_182919
31	small EDRK-rich factor 2 (SERF2),	NM_00101810 8
32	eukaryotic translation initiation factor 3, subunit E interacting protein (EIF3EIP),	NM_016091
33	guanine nucleotide binding protein (G protein), beta polypeptide 2-like 1 (GNB2L1),	NM_006098
