# Supplementary material for: Human cytomegalovirus UL23 inhibits transcription of interferon-γ stimulated genes and blocks antiviral interferon-γ responses by interacting with human N-myc interactor protein
Source: PLoS Pathog. 2018 Jan 29;14(1):e1006867. doi: 10.1371/journal.ppat.1006867 (PMC5805366; doi:10.1371/journal.ppat.1006867)
Supplement: S2 Table — Different cells were treated with IFN-γ (1000 U/ml) and then either mock-infected or infected with TowneBAC, ΔUL23, R-ΔUL23, UL23stop, or R-stop at 12 hours post-treatment. At 24 hours post-infection, we stained cells with DAPI, anti-UL23, anti-Nmi, or anti-STAT1, and visualized the cells under a microscope. The experimental procedures were described in Materials and Methods. (PDF) [file ppat.1006867.s003.pdf]

SUPPORTING INFORMATION

S2 Table. The percentages of the numbers of cells in which Nmi and STAT1 were found to be localized in the nuclei (nuclei), cytoplasm (cytoplasm), or both (nuclei/cytoplasm). Different cells were treated with IFN-γ (1000 U/ml) and then either mock-infected or infected with $Towne_{BAC}$, ΔUL23, R-ΔUL23, UL23stop, or R-stop at 12 hours post-treatment. At 24 hours post-infection, cells were stained with DAPI, anti-UL23, anti-Nmi, or anti-STAT1 respectively, and visualized using a microscope. The experimental procedures were described in Materials and Methods.

Expressed Protein/Cell(virus)	Nmi			STAT1		
Localization	Nuclei	Cytoplasm	Nuclei/Cytoplasm	Nuclei	Cytoplasm	Nuclei/Cytoplasm
U251	91±8%	1%	8%	92±8%	1%	7%
U251-FLAG	92±7%	1%	7%	90±9%	1%	9%
U251-C	91±9%	0%	9%	91±8%	0%	9%
U251-FLAG-UL23	0%	91±8%	9%	0%	92±9%	8%
U251-U23	0%	92±9%	8%	0%	91±8%	9%
U251 ($Towne_{BAC}$)	0%	93±7%	7%	0%	94±8%	6%
U251 (ΔUL23)	91±7%	1%	8%	92±8%	1%	7%
U251 (UL23stop)	91±8%	0%	9%	90±8%	1%	9%
U251 (R- ΔUL23)	0%	92±7%	8%	1%	91±9%	8%
U251 (R-stop)	0%	93±8%	7%	0%	92±7%	8%

The values shown are the means of three independent experiments. We examined 150 cells in each experiment, with a total of 450 cells in the three experiments. The values of SD that were less than 5% are not shown.
